# Evaluating Chinese large language models for HIV/AIDS health information: a multidimensional comparative study of response quality

**DOI:** 10.3389/fpubh.2026.1966522

**Published:** 2026-10-06

**Authors:** Jinhao Gu, Jianwei Liu, Yunrong Liu, Pingping Wang, Dehua Hu, Qizhen Zhu

**Affiliations:** 1 Department of Biomedical Informatics, School of Life Sciences, Central South University, Changsha, Hunan, China; 2 School of Medical Information Engineering, Jining Medical University, Rizhao, Shandong, China

**Keywords:** caring, digital health equity, health information quality, HIV/AIDS, large language models, latent profile analysis, partial correlation network

## Abstract

**Background:**

Chinese large language models (LLMs) are increasingly used for HIV/AIDS health information, yet their multidimensional performance remains unevaluated. This study systematically assessed five mainstream Chinese LLMs across accuracy, caring, completeness, understandability, and actionability.

**Methods:**

A cross-sectional evaluation was conducted using 50 standardized HIV/AIDS questions. Each model's responses were rated by three independent experts on five dimensions, including adapted PEMAT-based items. Latent profile analysis (LPA) and partial correlation network analysis were employed to identify quality typologies and interdimensional dependencies. Chi-square tests examined associations between profile membership and model identity or question domain.

**Results:**

The four general-purpose LLMs showed high accuracy (mean > 4.5/5) and completeness (86%−98% full marks), with no significant differences among them (all P-FDR > 0.05). HuatuoGPT-II, the only medical-domain-specific model, scored significantly lower on both dimensions (all *P* < 0.001). Its responses were less consistent with current guidelines and included one instance of implicit stigmatizing language. All models showed systemic deficits in caring (median = 3.0/5) and actionability (max = 1.70/3). Understandability showed a ceiling effect and no significant profile-level differences, although small model-level differences were observed. LPA identified four profiles: High Accuracy and Completeness (50.0%), Moderate-Balanced (3.6%), Low-Quality (18.8%), and High Completeness and Actionability-Oriented (27.6%). Network analysis identified completeness as the most central node (strength = 0.813) and actionability as a key bridge (betweenness = 0.167). A weak negative partial correlation between accuracy and actionability (*r* = −0.083) suggested a potential trade-off between technical precision and practical guidance at the statistical level. Profile distributions differed by model (χ^2^ = 124.99, *P* < 0.001) but not domain (*P* = 0.625), indicating heterogeneity stems from model-specific characteristics. Yuanbao-Hy3 showed the most balanced humanistic-technical profile. DeepSeek-V4 excelled technically but lacked caring.

**Conclusions:**

In this evaluation, the four general-purpose models outperformed the single medical-domain-specific model examined in factual accuracy and completeness. However, systemic deficits in empathetic communication and actionable guidance persist. Model selection should prioritize holistic profile distributions over single-dimension scores. Developers must integrate de-stigmatizing corpora and action-oriented alignment; clinicians should position AI as supplementary to professional care. Future evaluations should adopt person-centred, multi-turn, and real-user validated frameworks.

## 1 Introduction

Since the first AIDS case was reported in the United States in 1981, HIV/AIDS has remained a formidable global public health challenge (1). As of 2025, an estimated 40.9 million people worldwide are living with HIV, with approximately 1.2 million new infections and 570,000 AIDS-related deaths occurring each year. Over the past four decades, the global response has achieved substantial progress, with mortality declining steadily; however, this momentum is at risk of stalling, and without more decisive interventions, the goal of ending AIDS by 2030 will remain elusive (2). In China, although the overall prevalence of HIV and the number of AIDS-related deaths have long remained relatively low, the number of people living with HIV has surpassed one million, and new infections continue to rise (3). Meanwhile, the widespread adoption of antiretroviral therapy (ART) has significantly extended the life expectancy of people living with HIV, transforming the disease into a chronic condition requiring lifelong management and prolonged self-care (4). However, in China, inadequate public awareness of HIV, low health literacy and educational attainment among people living with HIV, and deeply entrenched social stigma converge to produce compounding adverse effects (3, 5, 6). These factors collectively contribute to weak preventive awareness and insufficient knowledge among susceptible populations, difficulties in effective self-management among those infected, persistent social discrimination and isolation, and, in extreme cases, retaliatory behavior by a small number of individuals living with HIV (7–9). Driven by stigma, convenience, and curiosity, individuals may turn to artificial intelligence (AI) to seek HIV-related information.

Large language models (LLMs) are built upon neural network architectures and are pre-trained on massive text corpora under self-supervised conditions, learning to predict the next token given preceding context or to fill in missing words based on surrounding text (10). Following pre-training, models are fine-tuned on task-specific labeled data to adapt the knowledge acquired during pre-training to the requirements of target tasks (11). This process endows models with the capacity to accurately comprehend questions, statements, and prompts. On this basis, models can generate contextually appropriate answers, suggestions, and explanations, and engage in coherent dialogue across a wide range of topics (12). Nevertheless, the outputs of LLMs are inherently probabilistic linguistic predictions rather than verified medical guidance, which raises questions about their credibility as a source of authoritative HIV-related advice (13). Despite this concern, models such as ChatGPT, DeepSeek, and Gemini—which can generate targeted text on demand at any time and place—have rapidly attracted widespread attention from HIV researchers and end-users alike (13–15). Tarhan and Sahin Ozdemir (13) compared the accuracy and reliability of ChatGPT-4o and Gemini in answering HIV-related questions and found that both models achieved high overall accuracy. De Vito et al. (14) evaluated the performance of ChatGPT in HIV prevention communication, noting that its responses were generally scientifically accurate but lacked inclusivity. Eren Korkmaz et al. (16) further compared three LLMs in terms of accuracy, readability, and reliability, demonstrating that Claude 3.7 Sonnet excelled in accuracy and reliability, while ChatGPT-4o produced more readable outputs.

Although existing studies have preliminarily explored the potential of LLMs in supplying HIV-related health information, three significant gaps remain in this field. First, current evaluations have largely been confined to technical dimensions such as accuracy and completeness, with scant attention paid to caring—a quality facet of paramount importance in the context of a highly stigmatized disease—thereby failing to capture the actual performance of AI outputs in terms of emotional support and de-stigmatizing expression. Second, prior studies have predominantly focused on foreign-developed models such as ChatGPT and Gemini, whose services have limited accessibility in mainland China and whose training corpora and cultural alignment are disconnected from local HIV prevention and control practices, thereby limiting the generalizability of their findings. Third, most studies have adhered to a variable-centred mean-comparison paradigm that presupposes sample homogeneity and linear additive relationships among dimensions, rendering them unable to identify configurational patterns and inherent dependency structures in AI response quality across a multidimensional space. The integrated framework of latent profile analysis (LPA) and partial correlation network analysis recently introduced by Deng et al. (17) in evaluating patient education texts on cardiac myxoma offers a methodological pathway for elucidating heterogeneous quality types and conditional associations among dimensions in AI-generated health content. Furthermore, given that people living with HIV in China generally exhibit low health literacy and educational attainment (3), a sole focus on content correctness is far from sufficient to ensure the practical utility of information; understandability and actionability are equally critical dimensions that determine the effectiveness of health education, yet they have been systematically overlooked in the existing literature. In light of these gaps, the present study draws on the analytical strategy of Deng et al. (17) to systematically evaluate five mainstream LLMs widely accessible in mainland China across five dimensions—accuracy, completeness, understandability, actionability, and caring—in response to HIV-related questions. By employing LPA to identify discrete quality profiles and partial correlation network analysis to delineate the conditional dependency structure among dimensions, aiming to move beyond unidimensional descriptive evaluation and characterize the configurational patterns and inter-dimensional associations of AI response quality as observed in the specific models tested, thereby providing more explanatory and actionable empirical evidence for the effective, safe, and equitable integration of artificial intelligence into HIV prevention, education, and practice in China.

## 2 Methods

### 2.1 Question selection and LLMs identification

Five LLMs were selected based on predefined inclusion criteria (Table 1). The four general-purpose models were required to simultaneously satisfy: (1) top-tier monthly active users in mainland China as of June 2026, or large-scale user reach through integration with leading platform ecosystems such as WeChat, Douyin, or Alipay (18); (2) free access to full conversational functionality via official public interfaces without professional deployment or paid subscription; (3) distinct developmental provenance and architectural lineage, ensuring coverage of the principal technology ecosystems of Chinese general-purpose LLMs; and (4) active version maintenance at the time of data collection (3 July 2026). Accordingly, DeepSeek-V4, Doubao 2.1, Qwen3.8-Max-Preview, and Yuanbao-Hy3 were included. To empirically test whether domain-specific models outperform general-purpose models in public-facing health education, HuatuoGPT-II was additionally included as a domain-specific comparator; this model is built upon the LLM4Med architecture and represents one of the most prominent medical-domain-specific Chinese LLMs (19). Fifty questions were drawn from two sources: the HIV health education repository developed by the National Center for AIDS/STD Control and Prevention, Chinese Center for Disease Control and Prevention (China CDC), and the Questions and Answers about HIV/AIDS published by the New York State Department of Health (3, 20). To ensure representativeness and methodological transparency, predefined inclusion and exclusion criteria were applied. Questions were included if they pertained to HIV diagnosis or management; were commonly encountered by the general public or people living with HIV (based on source frequency and senior clinician consultation); were unambiguously worded for consistent interpretation by human raters and LLMs; and required no access to personal medical records or real-time clinical data. Excluded were duplicate or highly similar questions and those not directly related to HIV/AIDS. Following independent screening of 92 candidate questions by two researchers with disagreements resolved via consensus with a third researcher, 86 questions were included. These were classified into five domains aligned with the HIV care continuum: basic knowledge, prevention (including PrEP/PEP), testing, treatment, and rehabilitation/care. Domain assignment was independently performed by two researchers and adjudicated by a senior infectious disease specialist. To ensure balance and minimize selection bias, 10 questions were randomly sampled from each domain via Python script, resulting in a final set of 50 questions (Supplementary Table S1). The allocation of 10 questions per domain was justified on three grounds. Statistically, this sample size provides ≥80% power to detect moderate-to-large effects (Cohen's *f* = 0.25) in mixed-effects models. Methodologically, equal distribution prevents overrepresentation of any single care stage, enabling valid cross-domain comparisons. Finally, comparable LLMs evaluation studies have demonstrated that similar or smaller per-domain samples suffice to capture stable inter-model differences while keeping expert rating burden feasible (21).

**Table 1:** Basic information and functional configurations of the selected large language models (as of 3 July 2026).

Attribute	DeepSeek-V4	Doubao 2.1	HuatuoGPT-II	Qwen3.8-Max-Preview	Yuanbao-Hy3
Architecture	Transformer	Transformer	Transformer	Transformer	Transformer
Interface	Web (chat.deepseek.com)	Web (doubao.com)	Web (huatuogpt.cn)	Web (tongyi.aliyun.com)	Web (yuanbao.tencent.com)
Version	Preview; undisclosed	doubao-seed-2-1-pro-260628	Undisclosed	Preview; undisclosed	Preview; undisclosed
Deep thinking	√	√	×	√	√
Web search	√	×	×	√	×
Generation parameters	Default	Default	Default	Default	Default

LLM, large language model; √, function available; ×, function unavailable.

All queries were submitted manually in Chinese through the official public web-based interfaces of each LLM to simulate the authentic experience of typical end-users. To ensure maximum consistency, all queries were administered within a strictly controlled time window (9:00 a.m. to 1:00 p.m. China Standard Time) on 3 July 2026. Prior to formal query submission, the precise version status for each model was confirmed via its platform interface: Doubao 2.1 bore the identifier doubao-seed-2-1-pro-260628; no public version identifiers were disclosed for Qwen3.8-Max-Preview, DeepSeek-V4, Yuanbao-Hy3, or HuatuoGPT-II at the time of query, with Qwen3.8-Max-Preview, DeepSeek-V4, and Yuanbao-Hy3 all remaining in preview phases. Furthermore, the functional configurations for each model were confirmed via interface settings prior to query submission: DeepSeek-V4 and Qwen3.8-Max-Preview had both “Deep Thinking” and “Web Search” functions enabled; Doubao 2.1 and Yuanbao-Hy3 had “Deep Thinking” enabled but “Web Search” was not available; and HuatuoGPT-II had neither function available.

To prevent carry-over effects from prior conversational context, a new chat session was initiated for each question by clicking the “New Chat” or equivalent function in the respective interface. During data collection, all models retained their public-facing default generation parameters (e.g., temperature, top-p), and no API-level or back-end parameter adjustments were made, ensuring that the evaluation assessed the “out-of-the-box” performance directly usable by users.

### 2.2 Assessment procedure and scoring criteria

Informed by the research gaps identified above, three raters who had received systematic training in HIV-related knowledge independently completed the scoring. The scoring criteria were grounded in the World Health Organization (WHO) publications Consolidated Guidelines on Differentiated HIV Testing Services, Overview of WHO Recommendations on HIV and STI Testing, Prevention, Treatment, Care and Service Delivery, WHO Updated Recommendations on HIV Clinical Management, and WHO Consolidated Operational Handbook on Sexually Transmitted Infections, as well as the National Technical Guidelines for HIV Testing (2025 Revision) issued by the China CDC and the Chinese Guidelines for Diagnosis and Treatment of HIV/AIDS (2024 Edition) published by the Society of Infectious Diseases, Chinese Medical Association (22–24, 37, 41–43). Five LLMs—DeepSeek-V4, Doubao 2.1, HuatuoGPT-II, Yuanbao-Hy3, and Qwen3.8-Max-Preview—were assessed across five dimensions: accuracy, completeness, caring, understandability, and actionability. Upon completion of independent scoring, the three raters convened a face-to-face consensus conference. Inter-rater agreement for all three raters was confirmed through consistency testing (Supplementary Tables S7–S11). For items on which ratings diverged, the raters jointly re-examined the corresponding LLMs responses and discussed them with reference to the aforementioned WHO and Chinese guidelines. Where disagreement persisted after deliberation, a senior infectious disease specialist who had not participated in the initial scoring was consulted to provide an independent professional opinion. Ultimately, through open discussion and iterative deliberation, all discrepancies were resolved, and each item received a single consensus integer score unanimously agreed upon by all three raters. These consensus scores were used in all subsequent statistical analyses. A five-point Likert scale was employed to rate accuracy, completeness, and caring (Supplementary Tables S2–S4). The Patient Education Materials Assessment Tool (PEMAT) (38) is a systematic instrument designed to evaluate and compare the understandability and actionability of patient education materials, with the aim of determining whether patients can comprehend the information and act upon it (25, 31). To accommodate the dynamic, text-dominant nature of LLM-generated responses, the original PEMAT underwent content-driven adaptation rather than psychometric re-validation. In the Understandability dimension (Supplementary Table S5), seven items were retained to address cognitive load risks from probabilistic generation: “Purpose evident” penalizes generic AI preambles; “Medical terms defined” mandates inline explanations to mitigate hyperlink hallucination risks; and “Visual cue effectiveness” was redefined to assess judicious use of text-based emphasis markers. Remaining general communication items received minimal wording adjustments; items lacking functional equivalents or redundant in conversational flows (e.g., visual aids, summaries) were excluded. In the Actionability dimension (Supplementary Table S6), three items were retained to bridge the gap between knowledge retrieval and behavioral guidance: “Direct address” requires second-person phrasing for personalized guidance; “Manageable steps” demands sequential instructions and penalizes vague quantifiers; and “Action identified” serves as a prerequisite filter for concrete behaviors. Items such as visual tools and external links were excluded due to interface constraints and hallucination risks. These adaptations establish content validity through structural alignment with LLM output features; formal psychometric validation remains uncompleted and is prioritized for future research. For ease of reference throughout the manuscript, PEMAT items were numbered sequentially: items 1–7 pertain to understandability, and items 8–10 pertain to actionability.

We applied Caring and Actionability scoring uniformly across all questions without domain-specific differentiation. This design rests on two principled grounds. First, in AI-mediated health communication for stigmatized diseases, avoiding depersonalizing or judgemental language constitutes a content-independent ethical safety baseline; Caring thus assesses whether a response causes implicit harm rather than demanding emotional counseling for every query. Second, even purely factual responses benefit from minimal contextual action cues (e.g., “consult an infectious disease specialist”), and Actionability captures this universal health-promoting function. Differentiating scoring by domain would introduce heterogeneity that undermines cross-question comparability and subsequent network validity. A uniform framework therefore ensures that the identified quality typologies reflect model-level generative characteristics.

### 2.3 Statistical analysis

Inter-rater reliability for ordinal ratings (accuracy, completeness, caring) was assessed using Krippendorff's alpha. For binary PEMAT items, Gwet's AC1 was used as the primary agreement statistic to avoid the kappa paradox caused by high prevalence, with Fleiss' kappa, KKrippendorff's alpha, prevalence-adjusted bias-adjusted kappa (PABAK), and percentage agreement reported for cross-validation.

For the five-level ordinal ratings, cumulative link mixed models (CLMMs) were fitted with LLM identity as a fixed effect and question identifier as a random intercept. Overall effects were evaluated via likelihood-ratio tests after verifying the proportional-odds assumption. *Post-hoc* pairwise comparisons were based on estimated marginal means, reporting odds ratios (ORs) and *P-values* adjusted by Holm, Bonferroni, and false discovery rate (FDR) corrections; significance was primarily determined using FDR-adjusted *P-values* and visualized via heatmaps. Sensitivity analyses employed permutation-based Friedman tests (10,000 permutations) and Kendall's W, followed by Wilcoxon signed-rank tests with Z-values and effect sizes (r). Pairwise conclusions from both approaches were compared to assess robustness.

For individual binary PEMAT items, overall differences were tested using Cochran's Q test, with *post-hoc* McNemar's tests (continuity-corrected) reporting χ^2^, OR, and corrected *P-values*. Skewed composite scores for understandability and actionability were analyzed using Friedman's test and Kendall's W, with Wilcoxon signed-rank *post-hoc* tests. Method selection was guided by data scale: CLMMs for ordinal repeated measures, permutation tests to circumvent asymptotic bias, Cochran's Q/McNemar for paired binary outcomes, and Friedman/Wilcoxon for skewed discrete data.

Latent profile analysis (LPA; *K* = 2–5) and partial correlation network analysis were conducted to examine multidimensional performance profiles. LPA model selection integrated Bayesian Information Criterion (BIC), Integrated Complete-data Likelihood (ICL), normalized entropy, minimum class proportion (>3%), and MinAvePP (>0.8); classification stability was evaluated via the adjusted Rand index (ARI) across 200 bootstrap resamples, with the optimal K defined as yielding the highest mean ARI while meeting predefined thresholds. Sparse partial correlation networks were estimated via Graphical Lasso with regularization selected through five-fold cross-validation; edges with |r| < 0.05 were pruned. Node centrality indices (strength, closeness, betweenness) were computed, and 1,000 non-parametric bootstrap iterations quantified stability (95% CI). Multinomial logistic regression was additionally fitted to predict LPA class membership from LLM identity and question. To evaluate the potential impact of shared question-level dependencies on the LPA and network findings—given that each of the 50 questions was answered by all five models—we conducted two supplementary robustness analyses. First, we generated de-meaned data by subtracting each question's mean (averaged across the five models) from the original scores for each dimension, thereby removing the question main effect, and re-estimated the LPA and network models. Second, we aggregated the data at the question level by taking the mean score across the five models for each question (*N* = 50), producing a fully independent dataset that naturally eliminates within-question dependencies, and repeated the LPA. The concordance between the original and robustness results was quantified using Pearson correlation for edge weights and Spearman's rank correlation for centrality rankings. Chi-square tests were used as an exploratory tool to examine profile–covariate associations, consistent with standard LPA practice. Given the modest size of one profile (3.6%, *n* = 9) and the clustered data structure (50 questions × 5 models), we supplemented the primary analysis with two robustness checks (de-meaning and question-level aggregation), which confirmed that the observed model–profile association was not materially affected by shared question-level variance. Nevertheless, the small class proportion warrants cautious interpretation, and replication in larger, independently sampled question banks is necessary to confirm profile stability.

## 3 Results

### 3.1 Descriptive statistics of the five dimensions

Inter-rater reliability analysis indicated acceptable to excellent agreement across all dimensions. For accuracy, completeness, and caring, Gwet's AC1 was ≥ 0.816 and KKrippendorff's alpha (ordinal) was ≥ 0.747; for understandability and actionability, Gwet's AC1 was ≥ 0.958 and ≥ 0.839, respectively, with Fleiss' kappa ≥ 0.821 and ≥ 0.822 (Supplementary Tables S7–S11).

Descriptive statistics for accuracy, completeness, and caring are presented in Figure 1 and Table 2. For accuracy, the four general-purpose models (DeepSeek-V4, Doubao 2.1, Qwen3.8-Max-Preview, and Yuanbao-Hy3) all achieved means above 4.5, with DeepSeek-V4 (4.70 ± 0.46) and Yuanbao-Hy3 (4.62 ± 0.49) performing best; HuatuoGPT-II scored markedly lower (4.06 ± 0.79) with greater dispersion. Completeness exhibited a pronounced ceiling effect: 86%−98% of responses from the four general-purpose models received full marks, compared with only 20% for HuatuoGPT-II (mean 3.64 ± 0.96). Caring scores were uniformly low, with a median of 3.0 across all models; DeepSeek-V4 (3.62 ± 0.75) and Yuanbao-Hy3 (3.64 ± 0.78) scored relatively higher, whereas HuatuoGPT-II was lowest (3.12 ± 0.39), with no response attaining a score of 5.

**Figure 1:**
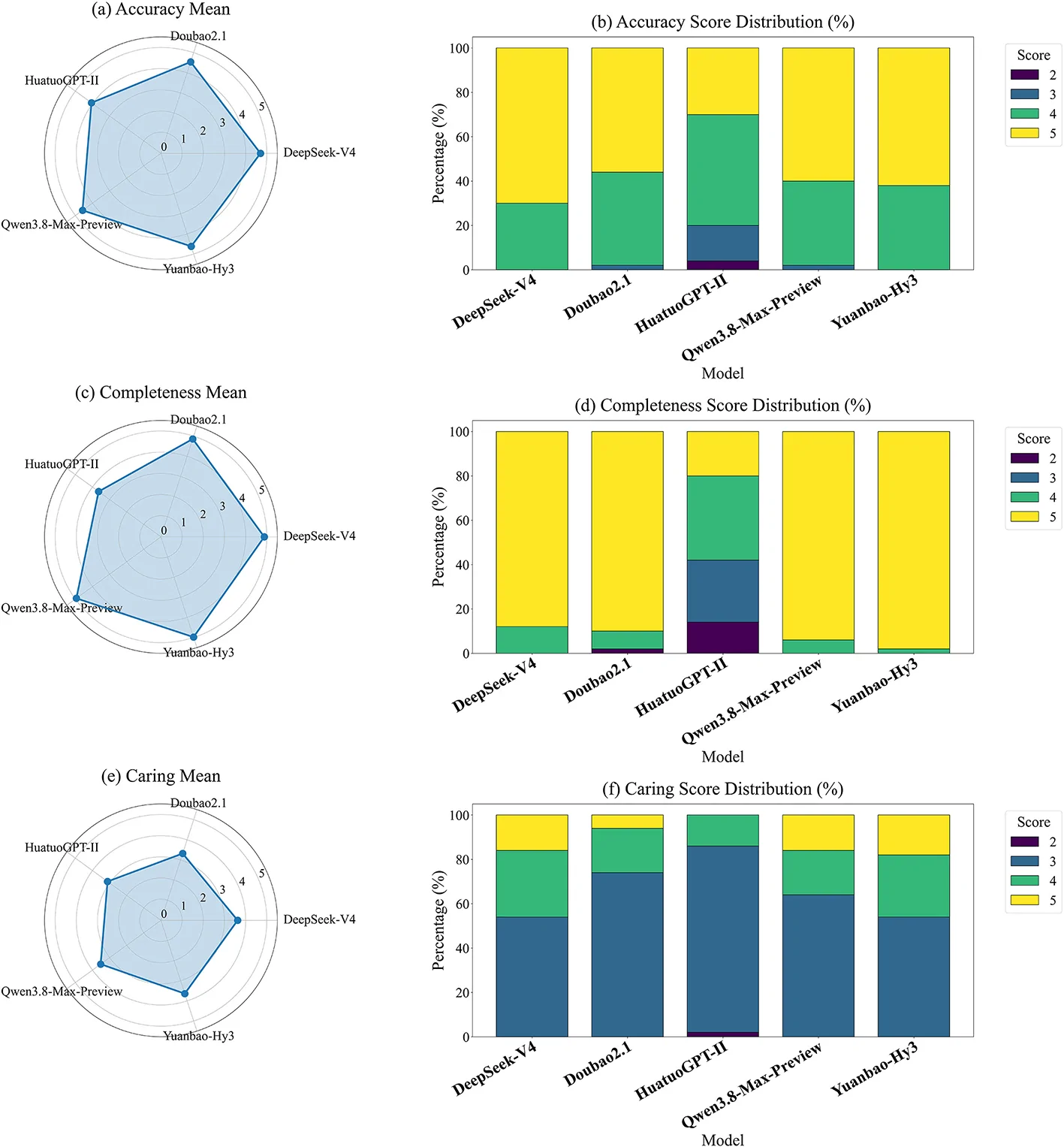
Descriptive statistics of accuracy, completeness, and caring scores by model. **(a)** Radar plot of mean accuracy scores. **(b)** Distribution of accuracy scores (percentage of responses at each score, 1–5). **(c)** Radar plot of mean completeness scores. **(d)** Distribution of completeness scores (percentage of responses at each score, 1–5). **(e)** Radar plot of mean caring scores. **(f)** Distribution of caring scores (percentage of responses at each score, 1–5). Left panels: radar plots of mean scores. Right panels: grouped bar charts of score distributions. Accuracy, completeness, and caring were rated on a 5-point Likert scale. Data are presented as mean ± SD.

**Table 2:** Descriptive statistics of accuracy, caring, and completeness scores by model.

Model	Dimension	Median (IQR)	Mean ±SD
DeepSeek-V4	Accuracy	5.0 (4.0–5.0)	4.70 ± 0.46
	Caring	3.0 (3.0–4.0)	3.62 ± 0.75
	Completeness	5.0 (5.0–5.0)	4.88 ± 0.33
Doubao 2.1	Accuracy	5.0 (4.0–5.0)	4.54 ± 0.54
	Caring	3.0 (3.0–3.75)	3.32 ± 0.59
	Completeness	5.0 (5.0–5.0)	4.86 ± 0.50
HuatuoGPT-II	Accuracy	4.0 (4.0–5.0)	4.06 ± 0.79
	Caring	3.0 (3.0–3.0)	3.12 ± 0.39
	Completeness	4.0 (3.0–4.0)	3.64 ± 0.96
Qwen3.8-Max-Preview	Accuracy	5.0 (4.0–5.0)	4.58 ± 0.54
	Caring	3.0 (3.0–4.0)	3.52 ± 0.76
	Completeness	5.0 (5.0–5.0)	4.94 ± 0.24
Yuanbao-Hy3	Accuracy	5.0 (4.0–5.0)	4.62 ± 0.49
	Caring	3.0 (3.0–4.0)	3.64 ± 0.78
	Completeness	5.0 (5.0–5.0)	4.98 ± 0.14

Accuracy, caring, and completeness were rated on a 5-point Likert scale. Data are presented as median (IQR) and mean ± SD; n = 50 responses per model. IQR, interquartile range; SD, standard deviation. Higher scores indicate better response quality.

Descriptive statistics for understandability and actionability are presented in Figure 2, Table 3. For understandability (total score 0–7), DeepSeek-V4 ranked highest (6.30 ± 0.86), followed by Qwen3.8-Max-Preview (5.92 ± 0.88) and Doubao 2.1 (5.86 ± 0.99), while HuatuoGPT-II was lowest (5.48 ± 1.18). Item-level analysis revealed uniformly high performance on “purpose evident” and “content focus” (pass rates ≥ 96%), but marked differences on “common language” and “medical terms defined”: DeepSeek-V4 explained medical terms in only 58% of responses, and Qwen3.8-Max-Preview in just 34%. Actionability (total score 0–3) was generally poor, with DeepSeek-V4 (1.70 ± 1.06) and Yuanbao-Hy3 (1.64 ± 0.96) performing relatively better, and HuatuoGPT-II (1.10 ± 0.79) and Doubao 2.1 (1.12 ± 0.82) weakest. Notably, the pass rate for “direct address” was only 4% for Doubao 2.1, and that for “manageable steps” was merely 8% for HuatuoGPT-II.

**Figure 2:**
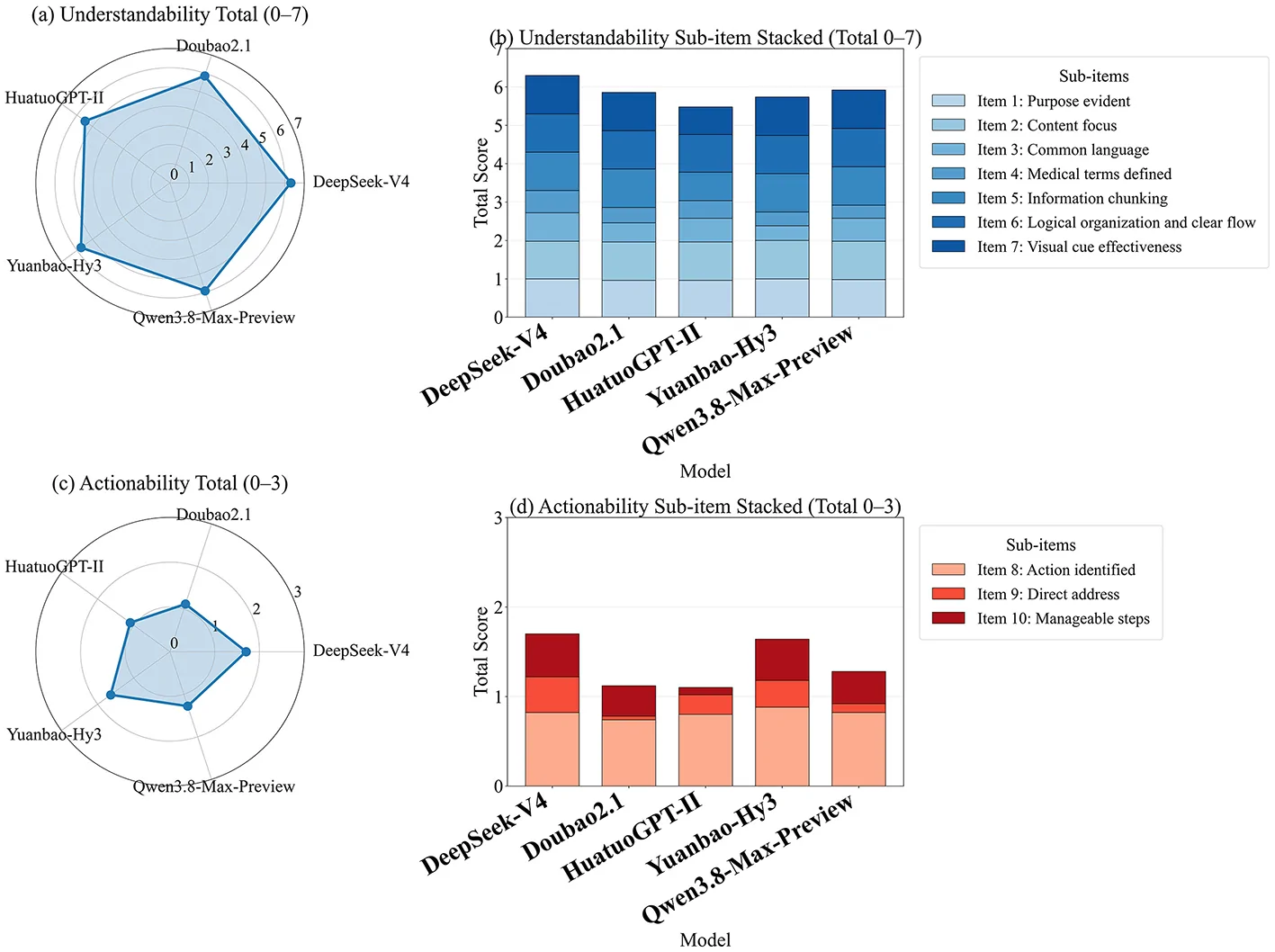
Descriptive statistics of understandability and actionability by model. **(a)** Radar plot of understandability total scores (0–7). **(b)** Stacked bar chart of item pass rates for understandability (7 items). **(c)** Radar plot of actionability total scores (0–3). **(d)** Stacked bar chart of item pass rates for actionability (3 items).

**Table 3:** Item-level pass rates and total scores for understandability and actionability by model.

Item/Total score	DeepSeek-V4	Doubao 2.1	HuatuoGPT-II	Qwen3.8-Max-Preview	Yuanbao-Hy3
Understandability					
Item 1: Purpose evident	1.00 (0.00)	0.96 (0.20)	0.96 (0.20)	0.98 (0.14)	1.00 (0.00)
Item 2: Content focus	0.98 (0.14)	1.00 (0.00)	1.00 (0.00)	1.00 (0.00)	1.00 (0.00)
Item 3: Common language	0.74 (0.44)	0.50 (0.50)	0.62 (0.49)	0.60 (0.49)	0.38 (0.49)
Item 4: Medical terms defined	0.58 (0.49)	0.40 (0.49)	0.46 (0.50)	0.34 (0.47)	0.36 (0.48)
Item 5: Information chunking	1.00 (0.00)	1.00 (0.00)	0.74 (0.44)	1.00 (0.00)	1.00 (0.00)
Item 6: Logical organization and clear flow	1.00 (0.00)	1.00 (0.00)	0.98 (0.14)	1.00 (0.00)	1.00 (0.00)
Item 7: Visual cue effectiveness	1.00 (0.00)	1.00 (0.00)	0.72 (0.45)	1.00 (0.00)	1.00 (0.00)
**Understandability Total (0–7)**	6.30 (0.86)	5.86 (0.99)	5.48 (1.18)	5.92 (0.88)	5.74 (0.92)
Actionability					
Item 8: Action identified	0.82 (0.38)	0.74 (0.44)	0.80 (0.40)	0.82 (0.38)	0.88 (0.33)
Item 9: Direct address	0.40 (0.49)	0.04 (0.20)	0.22 (0.41)	0.10 (0.30)	0.30 (0.46)
Item 10: Manageable steps	0.48 (0.50)	0.34 (0.47)	0.08 (0.27)	0.36 (0.48)	0.46 (0.50)
**Actionability Total (0–3)**	1.70 (1.06)	1.12 (0.82)	1.10 (0.79)	1.28 (0.86)	1.64 (0.96)

Values for individual items are means (SD) of binary pass/fail scores (0 or 1), where the mean represents the pass rate. Total scores are presented as mean (SD). Understandability total score ranges from 0 to 7; Actionability total score ranges from 0 to 3.

### 3.2 Pairwise comparisons among five LLMs

To assess inter-model differences, cumulative link mixed models (CLMMs) and permutation-based Friedman tests with Wilcoxon signed-rank *post-hoc* comparisons were used for accuracy, completeness, and caring. Under FDR correction, the significance decisions of the two approaches were fully concordant (Supplementary Table S18), although the two methods differ in their estimation frameworks (CLMM: model-based ordinal regression; Wilcoxon: rank-based non-parametric test). For these three dimensions, the reported *P*-FDR values are based on the CLMM *post-hoc* comparisons, with Wilcoxon results serving as a non-parametric sensitivity analysis. Cochran's Q tests (item-level) and Friedman tests (total scores) were used for understandability and actionability, all with FDR correction; for these two dimensions, the reported *P*-FDR values are based on Wilcoxon signed-rank *post-hoc* tests (total scores) or McNemar's tests (item-level). Detailed ORs, confidence intervals, effect sizes, and all adjusted *P-values* are provided in the Supplementary material.

For accuracy, CLMM and Wilcoxon results were highly consistent: HuatuoGPT-II scored significantly lower than all other models (all *P*-FDR < 0.001), with ORs ranging from 5.62 to 11.68; no significant differences were observed among the four general-purpose models (all *P*-FDR ≥ 0.215) (Figures 3a, b, Supplementary Tables S12, S13, S19, S20). For caring, HuatuoGPT-II again scored significantly lower than all other models (all *P*-FDR < 0.05); additionally, DeepSeek-V4 (OR = 3.87, *P*-FDR = 0.015) and Yuanbao-Hy3 (OR = 4.55, *P*-FDR = 0.007) significantly outperformed Doubao 2.1, while other pairwise comparisons were non-significant (Figures 3c, d, Supplementary Tables S14, S15, S19, S20). For completeness, HuatuoGPT-II was significantly inferior to all other models (all *P*-FDR < 0.001), with ORs reaching 52.29–360; the four general-purpose models did not differ significantly (all *P*-FDR ≥ 0.165), reflecting near-ceiling performance (Figures 3e, f, Supplementary Tables S16, S17, S19, S20). For understandability total scores, the Friedman test was significant (χ^2^ = 15.48, *P* = 0.004, Kendall's *W* = 0.077). *Post-hoc* Wilcoxon tests indicated that DeepSeek-V4 significantly outperformed HuatuoGPT-II (*P* = 0.001, *r* = 0.546), Yuanbao-Hy3 (*P* = 0.022, *r* = 0.403), and Doubao 2.1 (*P* = 0.047, *r* = 0.347); all other comparisons were non-significant (Figure 4a, Supplementary Table S21). At the item level, HuatuoGPT-II was significantly weaker than all other models on “information chunking” and “visual cue effectiveness” (all FDR *P* ≤ 0.002), with ORs of ∞ or 0, indicating entirely unidirectional discordance (Supplementary Table S22). For actionability total scores, the Friedman test was significant (χ^2^ = 26.67, *P* < 0.001, Kendall's *W* = 0.133). DeepSeek-V4 and Yuanbao-Hy3 did not differ significantly (*P* = 0.616), but both significantly outperformed Doubao 2.1, HuatuoGPT-II, and Qwen3.8-Max-Preview (all *P*-FDR ≤ 0.023; Figure 4b, Supplementary Table S23). On “direct address,” Doubao 2.1 performed worst (4% pass rate), significantly lower than HuatuoGPT-II and Yuanbao-Hy3; on “manageable steps,” HuatuoGPT-II had a pass rate of only 8%, significantly lower than all other models (all FDR *P* ≤ 0.015; Supplementary Table S23).

**Figure 3:**
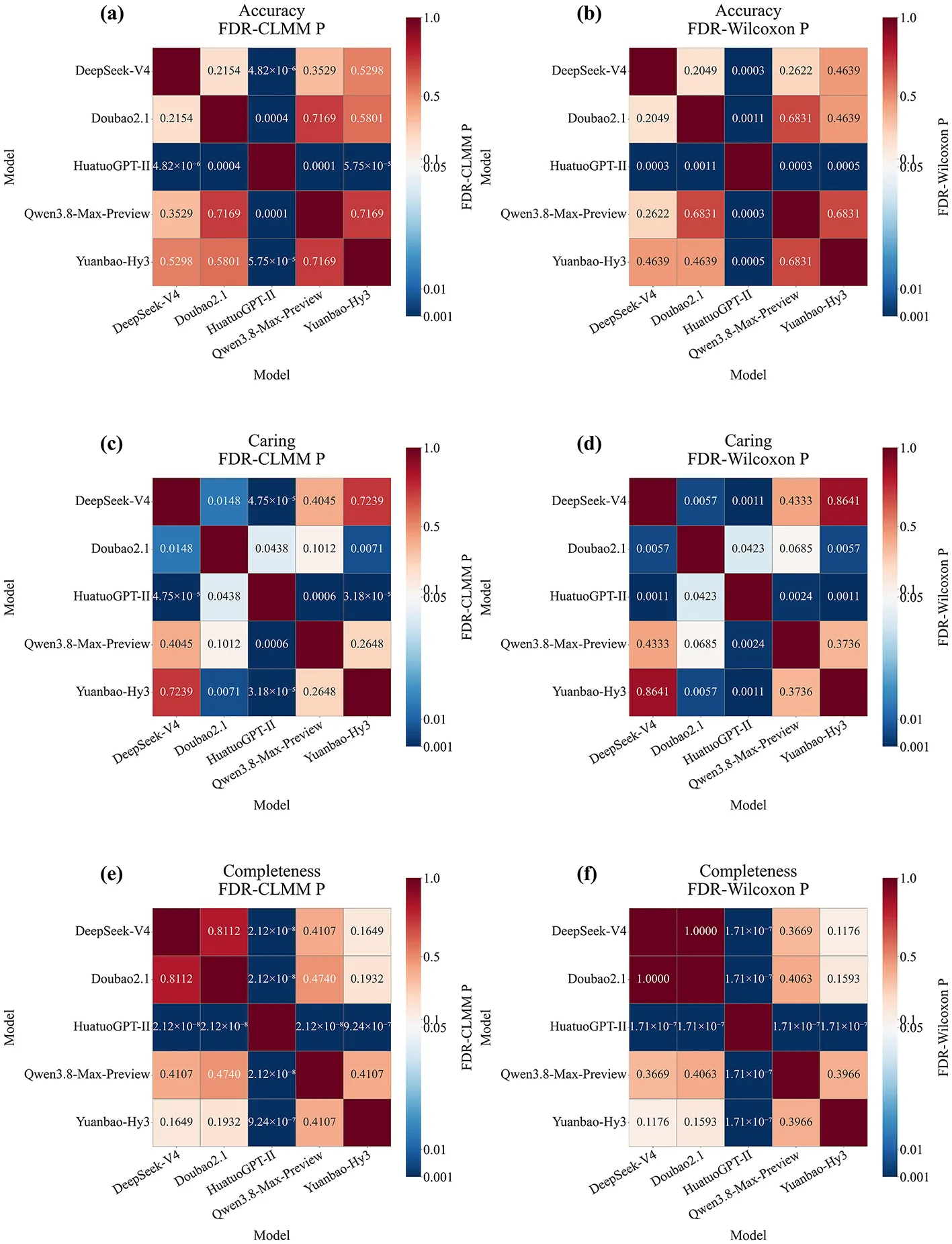
FDR-adjusted pairwise comparison heatmaps for accuracy, caring, and completeness. **(a, c, e)** CLMM *P-values*. **(b, d, f)** FDR-adjusted *P-values*. Pairwise comparisons among five models (DeepSeek-V4, Doubao 2.1, HuatuoGPT-II, Qwen3.8-Max-Preview, Yuanbao-Hy3). Color scale indicates *P*-*value*, with red denoting significant and blue denoting non-significant differences. Detailed odds ratios and confidence intervals are provided in Supplementary Tables S12–S17, S19, S20.

**Figure 4:**
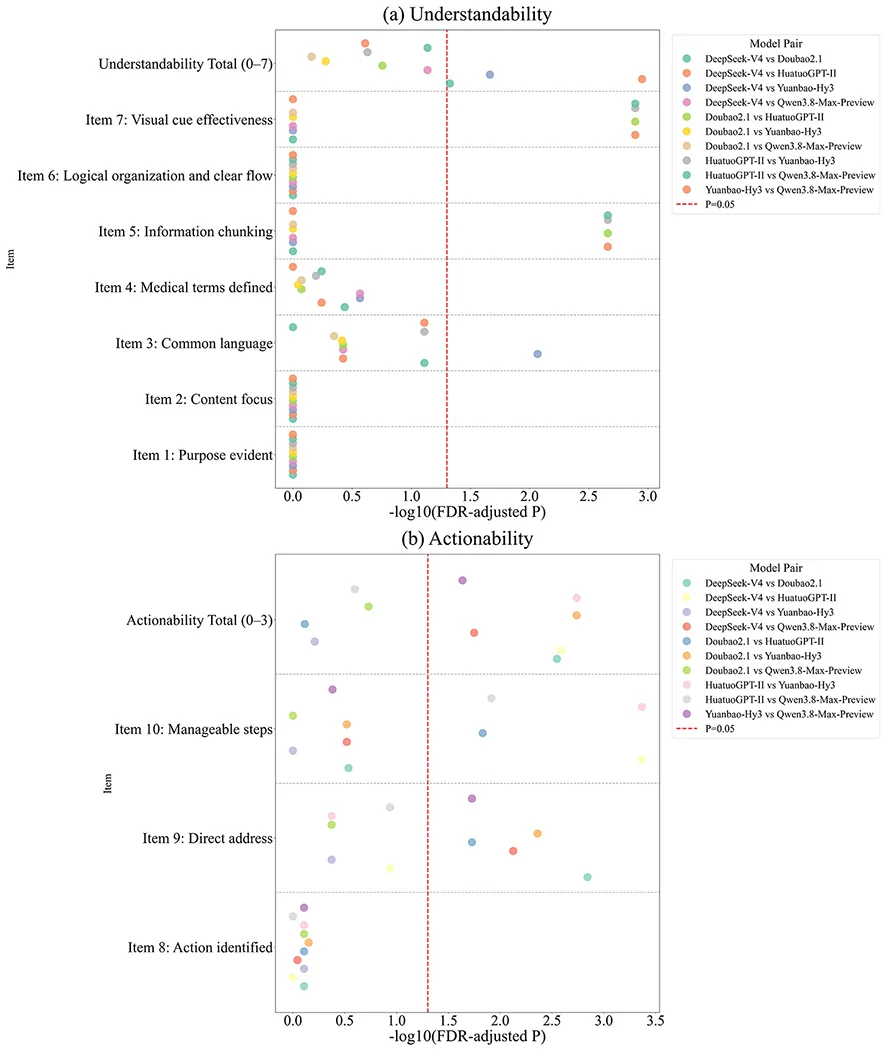
Dot plots of pairwise comparisons for understandability and actionability. **(a)** Understandability items and total score. **(b)** Actionability items and total score. The x-axis represents –log10 (FDR-adjusted *P*). The red dashed line indicates *P* = 0.05. Each dot represents one model pair. Detailed ORs and effect sizes are provided in Supplementary Tables S21 and S23.

Under the three correction methods (FDR, Holm, and Bonferroni), the overall patterns of differences across the five dimensions were largely consistent, although the significance of some comparisons changed under more stringent corrections. For accuracy and completeness, all differences involving HuatuoGPT-II remained significant under all three corrections (all *P* < 0.001), indicating robust conclusions. For caring, the difference between Doubao 2.1 and HuatuoGPT-II was significant under FDR correction (*P*-FDR = 0.044) but lost significance under Holm (*P* = 0.131) and Bonferroni (*P* = 0.263), suggesting a weak effect that warrants cautious interpretation. For understandability, the total score difference between DeepSeek-V4 and Doubao 2.1 was significant under FDR correction (*P*-FDR = 0.047) but not under Holm (*P* = 0.113) or Bonferroni (*P* = 0.142), likewise requiring caution. For actionability, some comparisons among general-purpose models (e.g., DeepSeek-V4 vs. Qwen3.8-Max-Preview) did not remain significant under Bonferroni correction, indicating sensitivity to the correction method. Overall, the inferiority of HuatuoGPT-II was robust across all corrections, whereas subtle differences among general-purpose models should be interpreted with caution, taking effect sizes and correction methods into account. Results under Holm and Bonferroni corrections are provided in Supplementary Tables S19, S20 (accuracy, completeness, caring), Supplementary Table S22 (understandability), and Supplementary Table S24 (actionability), respectively.

### 3.3 Determination of the optimal number of latent profiles and model stability verification

In terms of information criteria, both BIC and ICL decreased monotonically as the number of classes increased (*K* = 2: BIC = 1,874.06, *K* = 3: 361.35, *K* = 4: 93.40, *K* = 5: −242.59), indicating that the five-class solution achieved the best statistical goodness-of-fit. However, model selection should not rely solely on absolute fit indices but must also account for classification stability and clinical interpretability. Bootstrap resampling analysis (200 iterations) revealed that the four-class solution yielded the highest mean adjusted Rand index (ARI = 0.823, 95% CI: 0.329–1.000), with a median of 0.945, demonstrating superior reproducibility of class assignments under resampling. By contrast, although the five-class solution was marginally superior in BIC, its mean ARI was only 0.595 (median = 0.607), suggesting unstable classification boundaries under perturbation and a risk of over-extraction. The mean ARIs for *K* = 2 and *K* = 3 were 0.656 and 0.754, respectively, both lower than that of *K* = 4. Regarding classification quality, normalized entropy exceeded 0.99 and minimum average posterior probability (MinAvePP) exceeded 0.99 for all four candidate solutions, indicating excellent classification clarity. The smallest class proportion for *K* = 4 was 3.6% (*n* = 9), slightly below the conventional 5% threshold. However, this subgroup exhibited a distinctive profile pattern in dimensional means and carried independent theoretical significance, and was therefore retained with the explicit caveat that its stability requires verification in larger samples. Integrating goodness-of-fit, bootstrap stability, and interpretability, *K* = 4 was selected as the optimal latent profile solution (Table 4, Figures 5b–d).

**Table 4:** Fit indices and bootstrap stability for latent profile analysis models (*K* = 2–5).

K	BIC	ICL	Entropy	Min Class %	MinAvePP	ARI (Mean)	ARI (Median)	ARI 95% CI
2	1,874.06	1,875.17	0.997	22.0	1.000	0.656	1.000	[0.151, 1.000]
3	361.35	361.38	0.999	22.4	1.000	0.754	0.907	[0.282, 1.000]
4	93.40	94.92	0.998	3.6	0.999	0.823	0.945	[0.329, 1.000]
5	−242.59	−241.16	0.998	3.6	0.999	0.595	0.607	[0.285, 0.936]

BIC, Bayesian Information Criterion; ICL, Integrated Complete-data Likelihood; AvePP, Average Posterior Probability; ARI, Adjusted Rand Index from 200 bootstrap resamples. Although K = 5 yielded the lowest BIC/ICL, its bootstrap stability (ARI = 0.595) was substantially lower than K = 4 (ARI = 0.823). K = 4 was selected as the optimal solution based on the combination of highest stability, adequate entropy (>0.99), and interpretability. Min Class % = 3.6% (n = 9) for K = 4 represents a small but clinically meaningful subgroup.

**Figure 5:**
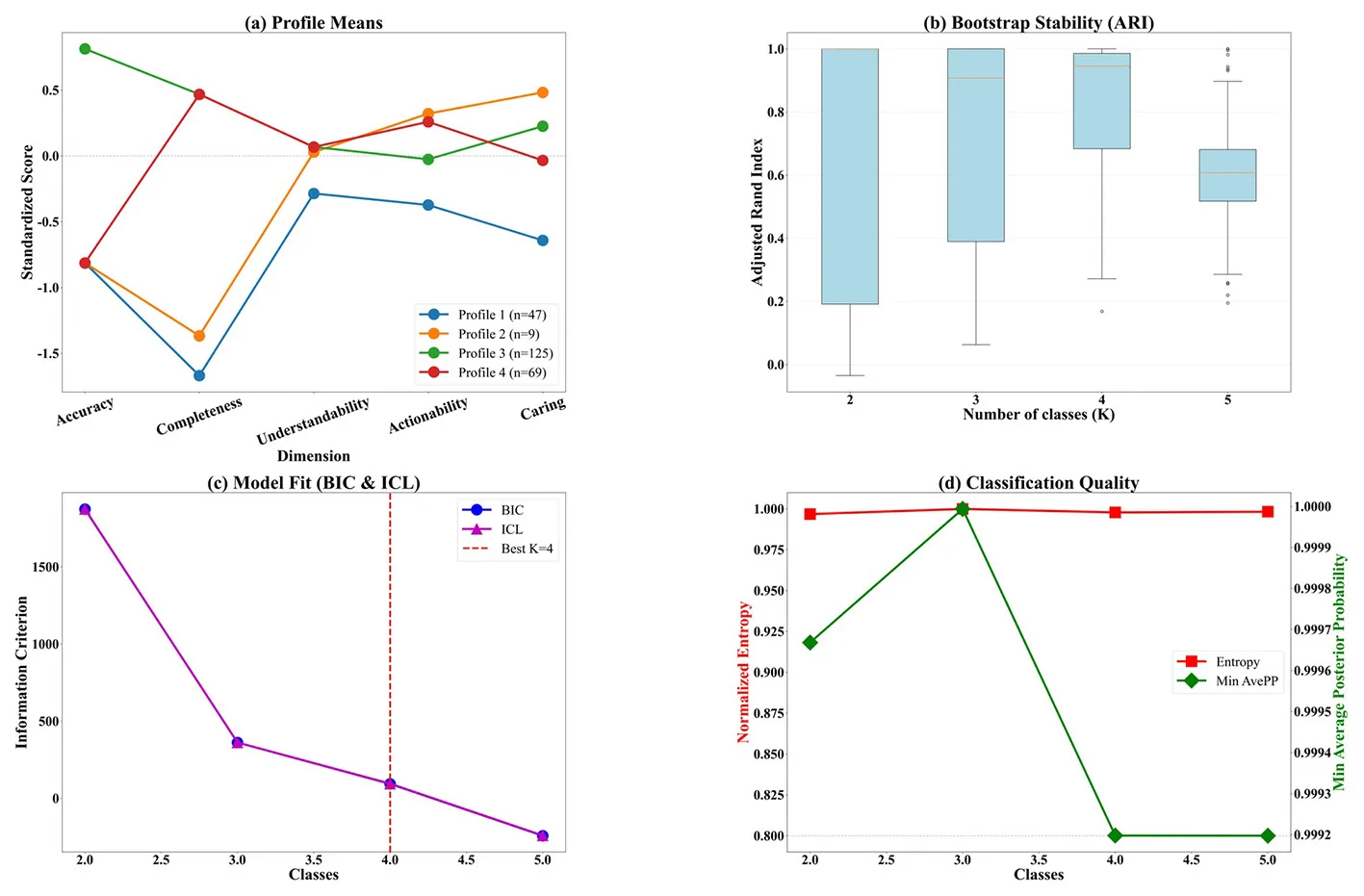
Latent profile analysis of AI response quality (*N* = 250). **(a)** Standardized mean scores across the five dimensions for the four profiles. **(b)** Bootstrap stability (adjusted Rand index; ARI) across 200 resamples. **(c)** Model fit (BIC and ICL) across *K* = 2–5, with the optimal *K* = 4 marked by a red dashed line. **(d)** Classification quality (normalized entropy and MinAvePP). Detailed results are provided in Table 5, and Supplementary Table S25.

### 3.4 Characterization of four latent profiles and between-group comparisons

Means, standard deviations, and between-group test results for each profile are presented in Table 5; standardized score line plots are shown in Figure 5a.

**Table 5:** Dimension-level mean scores of the four latent profiles of AI-generated HIV/AIDS response quality.

Variable	Profile 1	Profile 2	Profile 3	Profile 4
Accuracy	4.00 ± 0.78	4.00 ± 0.71	5.00 ± 0.00	4.00 ± 0.00
Caring	3.00 ± 0.00	3.78 ± 0.67	3.60 ± 0.77	3.42 ± 0.65
Completeness	3.45 ± 0.77	3.67 ± 0.71	5.00 ± 0.00	5.00 ± 0.00
Understandability	0.80 ± 0.18	0.84 ± 0.13	0.85 ± 0.14	0.85 ± 0.13
Actionability	0.34 ± 0.27	0.56 ± 0.29	0.45 ± 0.33	0.54 ± 0.28

Values are mean ± SD. Accuracy, caring, and completeness were rated on a 5-point Likert scale; understandability total score ranges from 0 to 7; actionability total score ranges from 0 to 3. N = 250 model–question responses.

Profile 1: Low-Quality (*n* = 47, 18.8%) exhibited the lowest scores across all dimensions: accuracy (4.00 ± 0.78), completeness (3.45 ± 0.77), understandability (0.80 ± 0.18), actionability (0.34 ± 0.27), and caring (3.00 ± 0.00). The zero standard deviation for caring indicates uniformly minimal humanistic engagement, constituting a “globally deficient” pattern.

Profile 2: Moderate-Balanced (*n* = 9, 3.6%) displayed moderate accuracy (4.00 ± 0.71) and completeness (3.67 ± 0.71) but the highest caring (3.78 ± 0.67) and actionability (0.56 ± 0.29), representing “moderate technical quality with prominent humanistic caring.” However, this subgroup represents only a small fraction of the overall sample; its distinctive mean profile, although theoretically meaningful, should be interpreted provisionally given the modest class size and the exploratory nature of LPA. Replication in larger and independently drawn question sets is necessary to establish whether this pattern reflects a stable response type or a sampling artifact.

Profile 3: High Accuracy and Completeness (*n* = 125, 50.0%) achieved maximum accuracy and completeness (both 5.00 ± 0.00) with zero variance, yet only moderate caring (3.60 ± 0.77) and actionability (0.45 ± 0.33), exhibiting a “high-tech, low-warmth” pattern.

Profile 4: High Completeness and Actionability-Oriented (*n* = 69, 27.6%) exhibited perfect completeness (5.00 ± 0.00), the highest actionability among all four profiles (0.54 ± 0.28), moderate accuracy (4.00 ± 0.00), and a caring score of 3.42 ± 0.65, which was marginally lower than that of Profile 3 (3.60). This profile is therefore best characterized as emphasizing comprehensive information delivery and practical behavioral guidance, while accepting modest trade-offs in factual precision and affective tone relative to the technically optimal Profile 3. Notably, Profile 2 (Moderate-Balanced) attained the highest caring score (3.78), indicating that empathetic engagement is not the defining feature of Profile 4.

Between-group comparisons confirmed significant differences among profiles in accuracy (*F* = 160.16, *P* < 0.001; H = 194.75, *P* < 0.001), completeness (*F* = 260.61, *P* < 0.001; H = 239.53, *P* < 0.001), actionability (*F* = 4.20, *P* = 0.006; H = 13.30, *P* = 0.004), and caring (*F* = 10.29, *P* < 0.001; H = 33.23, *P* < 0.001). Understandability was the sole non-significant dimension (*F* = 1.58, *P* = 0.194; H = 2.64, *P* = 0.451), with means clustered within 0.80–0.85 across all profiles, indicating a pronounced ceiling effect and homogeneity in linguistic readability.

### 3.5 Partial correlation network structure and centrality analysis

A partial correlation network was estimated using Graphical Lasso (λ selected via five-fold cross-validation; edges with |partial r| < 0.05 pruned). Network topology, edge weights, and centrality indices are presented in Figures 6a, c, Table 6, and Supplementary Table S26.

**Figure 6:**
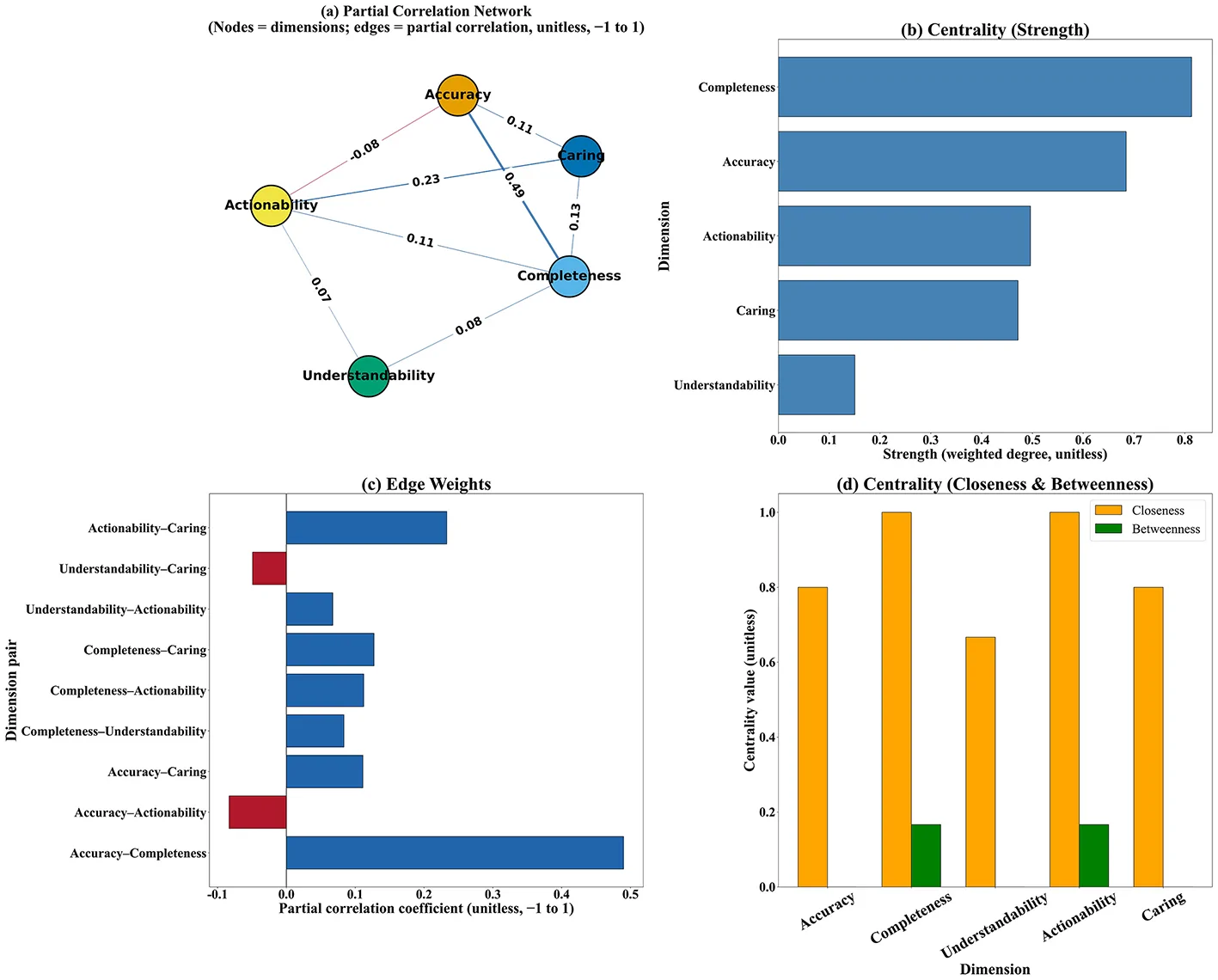
Partial correlation network of evaluation dimensions. **(a)** Regularized partial correlation network (Graphical Lasso CV). Blue/red edges indicate positive/negative associations; only *r* ≥ 0.05 shown. Strongest edge: Accuracy–Completeness (*r* = 0.49). **(b)** Strength centrality; Completeness was highest (0.81). **(c)** Edge weights for all pairs. **(d)** Closeness and betweenness centrality indices.

**Table 6:** Partial correlation network centrality indices among five response quality dimensions.

Dimension	Strength	Closeness	Betweenness
Accuracy	0.685	0.800	0.000
Completeness	0.813	1.000	0.167
Understandability	0.151	0.667	0.000
Actionability	0.496	1.000	0.167
Caring	0.472	0.800	0.000

Network estimated via Graphical Lasso with 5-fold cross-validated λ; edges with |partial r| < 0.05 thresholded to zero. Strength, sum of absolute edge weights; Closenes, inverse average shortest path; Betweenness, proportion of shortest paths passing through the node.

Completeness exhibited the highest strength (0.813) and perfect closeness (1.000), identifying it as the most influential dimension for overall response quality. Actionability and Completeness shared the highest betweenness (0.167), serving as bridge nodes connecting technical quality (Accuracy–Completeness cluster) to humanistic quality (Caring). Understandability was the most peripheral node (strength = 0.151), consistent with the non-significant profile differences in Supplementary Table S25.

The sparse network retained eight valid edges. The strongest edge connected accuracy and completeness (partial *r* = 0.490), forming a “technical quality cluster.” The second-strongest linked actionability and caring (*r* = 0.233), constituting a “humanistic-practical cluster.” A negative partial correlation between accuracy and actionability (r = −0.083) suggested that, after controlling for other dimensions, excessive technical focus may slightly suppress practical actionability.

Centrality analysis (Table 6; Figures 6b, d) identified completeness as the topologically most central node, ranking highest or jointly highest in strength (0.813), closeness (1.000), and betweenness (0.167). Actionability shared the highest betweenness (0.167) and matched completeness in closeness (1.000), positioning it as a statistical connector between the technical and humanistic-practical clusters within the conditional dependence network. Importantly, these centrality indices quantify the strength and breadth of partial correlations among dimensions rather than indicating causal priority or directional intervention effects (26, 27).

Completeness exhibited the highest strength (0.813) and perfect closeness (1.000), identifying it as the most influential dimension for overall response quality. Actionability and Completeness shared the highest betweenness (0.167), serving as bridge nodes connecting technical quality (Accuracy–Completeness cluster) to humanistic quality (Caring). Understandability was the most peripheral node (strength = 0.151), consistent with the non-significant profile differences in Supplementary Table S25.

### 3.6 Distribution of latent profiles across AI models and question domains

Cross-tabulations of model × profile and domain × profile are presented in Table 7, with row-percentage distributions visualized as stacked bar charts in Figure 7. Chi-square testing revealed a highly significant association between model identity and profile membership (χ^2^ = 124.99, *df* = 12, *P* < 0.001). Specifically, 68.0% of HuatuoGPT-II responses fell into Profile 1 (vs. 2.0%−10.0% for the other models), indicating generally low response quality. Yuanbao-Hy3 showed the most balanced performance, with 38.0% of responses in Profile 4 and only 2.0% in Profile 1. DeepSeek-V4 had 64.0% in Profile 3, reflecting outstanding technical accuracy but insufficient humanistic caring. Qwen3.8-Max-Preview and Doubao 2.1 exhibited similar distributions, with substantial proportions in both Profiles 3 and 4 (58.0%/34.0% and 54.0%/36.0%, respectively) and ≤ 10.0% in Profile 1.

**Table 7:** Distribution of latent profiles across AI models and question domains.

Category	Subgroup	Profile 1 (%)	Profile 2 (%)	Profile 3 (%)	Profile 4 (%)	*χ^2^* (*df* = 12)	*P*
AI model	DeepSeek-V4	10.0	2.0	64.0	24.0	124.99	< 0.001
	Doubao 2.1	10.0	0.0	54.0	36.0		
	HuatuoGPT-II	68.0	12.0	14.0	6.0		
	Qwen3.8-Max-Preview	4.0	4.0	58.0	34.0		
	Yuanbao-Hy3	2.0	0.0	60.0	38.0		
Question domain	AIDS basic knowledge	22.0	0.0	60.0	18.0	9.90	0.625
	Prevention	18.0	4.0	50.0	28.0		
	Rehabilitation & care	16.0	4.0	52.0	28.0		
	Testing	20.0	8.0	40.0	32.0		
	Treatment	18.0	2.0	48.0	32.0		

Values are row percentages within each subgroup; n = 50 responses per subgroup. Profile 1 = Low-Quality; Profile 2 = Moderate-Balanced; Profile 3 = High Accuracy and Completeness; Profile 4 = High Completeness and Actionability-Oriented. χ^2^ tests assessed whether profile membership differed across models or domains. Model × Profile association was highly significant (χ^2^ = 124.99, df = 12, P < 0.001), with HuatuoGPT-II showing the highest proportion of low-quality responses and Yuanbao-Hy3 the highest proportion of comprehensive–humanistic responses. Domain × Profile association was not significant (χ^2^ = 9.90, df = 12, P = 0.625), indicating that profile membership was driven primarily by model-specific generative characteristics rather than question content area.

**Figure 7:**
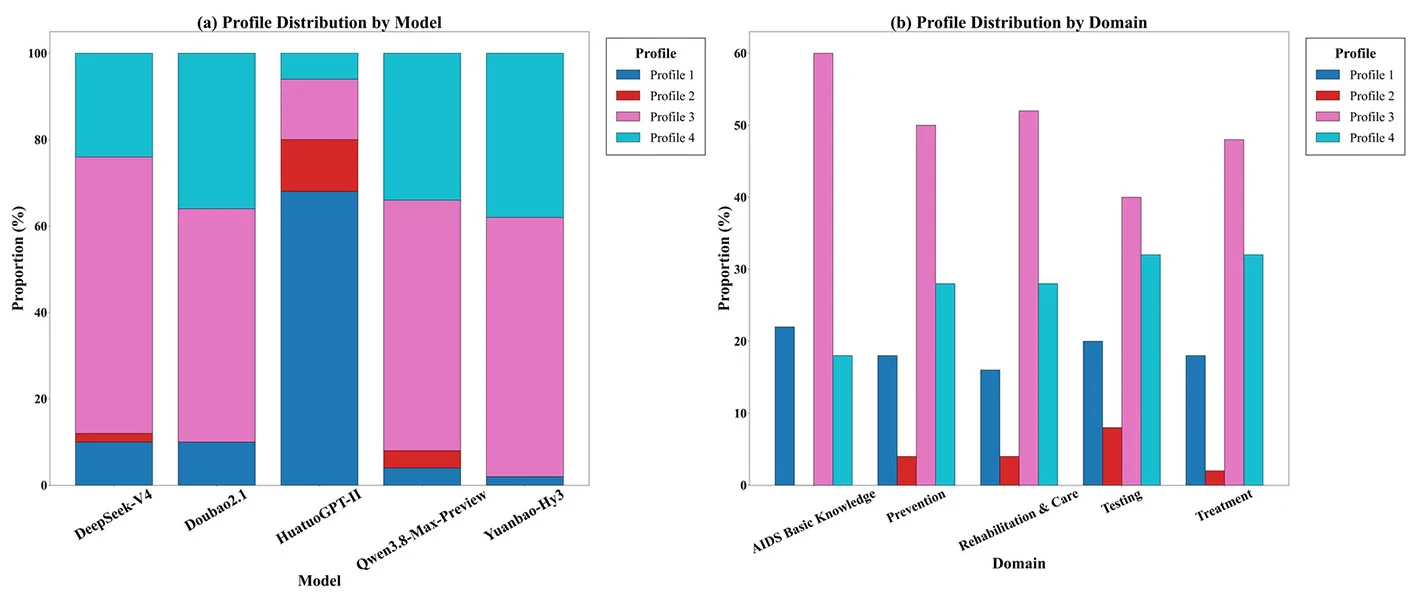
Distribution of latent profiles across models and question domains. **(a)** Profile distribution by model (row percentages). **(b)** Profile distribution by question domain (row percentages). Model identity was significantly associated with profile membership (χ^2^ = 124.99, *df* = 12, *P* < 0.001), whereas question domain was not (χ^2^ = 9.90, *df* = 12, *P* = 0.625). Numerical values are provided in Table 7.

In contrast, no significant association was found between question domain and profile membership (χ^2^ = 9.90, *df* = 12, *P* = 0.625). Profile distributions were consistent across all five domains: Profile 3 accounted for 40.0%−60.0% of responses, while Profile 1 ranged from 16.0% to 22.0%. These findings indicate that heterogeneity in AI response quality is primarily driven by model-specific generative characteristics rather than by the medical domain of the question.

The robustness checks confirmed that shared question-level structure did not materially affect our primary conclusions (in Supplementary Table S27). In the de-meaned data, LPA recovered four morphologically identical profiles (entropy > 0.99; MinAvePP > 0.99), and the network showed excellent concordance with the original (edge weights: *r* = 0.942; Strength centrality: ρ = 0.900). Conversely, question-level aggregation (*N* = 50) yielded only a single-class solution (*K* = 1), corroborating the non-significant domain–profile association (χ^2^ = 9.90, *P* = 0.625). Collectively, these results demonstrate that observed heterogeneity is driven predominantly by model-specific characteristics rather than question content.

## 4 Discussion

This study presents a systematic, multidimensional evaluation of five Chinese LLMs in responding to HIV/AIDS-related questions within a standardized, 50-question framework. This is the first to apply latent profile analysis and partial correlation network analysis to characterize heterogeneity and conditional dependence structures in AI-generated health information for a highly stigmatized infectious disease. These findings should be interpreted within the boundaries of the present design. They are conditioned by the specific models examined, the fixed question bank, and the single-turn query format. Accordingly, they are best regarded as exploratory evidence mapping the current performance landscape of a selected group of Chinese LLMs, rather than as definitive or broadly generalizable statements about all such systems or all HIV/AIDS information contexts. Since the advent of ChatGPT, LLMs have emerged as a novel channel for public health information, particularly in sensitive domains like HIV/AIDS where stigma deters open discussion (34, 51). However, existing literature has predominantly focused on Western models and technical metrics such as accuracy and completeness, neglecting humanistic and practical dimensions including caring, understandability, and actionability (15, 36, 51, 52). Moreover, these models are not routinely accessible in mainland China. Given China's conservative cultural context and the substantial population of people living with HIV/AIDS (PLWHA) who may seek anonymous information due to shame (39, 48), evaluating domestic LLMs across five dimensions—accuracy, caring, completeness, understandability, and actionability—is critical to preventing misinformation, stigma reinforcement, and disruption of patient–provider relationships.

Regarding accuracy, general-purpose LLMs demonstrated high performance, with DeepSeek-V4 achieving a 70% perfect accuracy rate. No statistically significant differences were observed among the four general-purpose models (adjusted *P* ≥ 0.215), suggesting convergence in factual knowledge retrieval under current pre-training paradigms (Zhao et al., 2023; Tarhan et al., 2024). In contrast, HuatuoGPT-II, the only medical-domain-specific model, scored significantly lower than all general-purpose models (all *P* < 0.001). This observed discrepancy may be related to differences in the training data and information access characteristics of the evaluated models. HuatuoGPT-II's responses suggest a reliance on structured clinical literature and guidelines that may not reflect the most recent recommendations (36, 51), as illustrated by its suggestion of “re-testing at week 24,” which is inconsistent with current World Health Organization (22) and Chinese national guidelines (23), instead conveying outdated information. By contrast, the four general-purpose models evaluated in this study appeared to reflect more current information, though we did not directly examine their training data composition, retrieval mechanisms, or knowledge-update processes. Importantly, the single medical-domain-specific model did not prioritize practical guidance to the same degree as the general-purpose models; however, this observation pertains to the specific model tested and should not be generalized to all medical-domain-specific models. It is equally important to note that only one medical-domain-specific model was evaluated. The observed differences should therefore be attributed to the particular model tested, and should be extrapolated with caution to medical-domain-specific or general-purpose models as categories.

The caring dimension revealed a systemic deficit across all models. The median score was only 3.0, far below accuracy and completeness scores. Even the best-performing models (DeepSeek-V4 and Yuanbao-Hy3) achieved a maximum mean of only 3.62/3.64, indicating a predominantly neutral tone. This resonates cross-culturally with De Vito et al. (14), who found ChatGPT's HIV prevention communication “scientifically accurate but insufficiently inclusive,” suggesting the deficit is systemic rather than model-specific. Notably, HuatuoGPT-II received a rating of 2 for one response containing implicit bias: when explaining the “ABC principle,” it interpreted “A (Abstinence)” as “prohibiting non-marital sexual behavior” rather than simply “abstinence,” implying moral judgement. While our study cannot determine the precise origin of this phrasing, one plausible hypothesis is that it reflects lingering traces of outdated health education materials present in the model's training corpus. This phrasing likely originates from outdated health education materials in its training corpus. In China's socio-cultural context where People living with HIV/AIDS (PLWHA) face moral prejudice (48), such discriminatory undertones risk reinforcing internalized stigma and reducing willingness to seek care (32, 35, 47). Deficiencies in caring are thus substantive ethical flaws, not merely stylistic issues. We urge developers to incorporate empathetic expression and de-stigmatizing corpora during alignment training and to update corpora promptly to eliminate stigmatizing expressions, rather than treating factual correctness as the sole optimization objective. Based on these observational findings, we hypothesize that incorporating empathetic expression and de-stigmatizing corpora during alignment training, alongside timely updates to purge stigmatizing content, could mitigate such risks. However, this hypothesis remains to be empirically tested in controlled intervention studies, and we offer it as a directional suggestion for future model development rather than a conclusion established by our current output-level analysis.

Completeness exhibited the most pronounced polarization. The 86%−98% of responses from general-purpose models received full marks, compared to only 20% for HuatuoGPT-II. This aligns with Xin et al. (44), who found HuatuoGPT-II scored significantly lower than DeepSeek-V4 in completeness for blepharoplasty consultation, suggesting this limitation may be common to medical-domain-specific models in public-facing Q&A. The mechanism likely involves general-purpose models' exposure to broad popular health texts and encyclopedias during pre-training, enabling multidimensional background knowledge supplementation, whereas HuatuoGPT-II's focus on specialized guidelines and conservative reward mechanisms favor concise declarative facts over breadth. From a public health perspective, high completeness enables comprehensive information provision in single interactions, reducing cognitive bias from fragmented information. However, caution is warranted regarding potential information overload for PLWHA with lower educational attainment, as lengthy responses may reduce core information accessibility (49), an issue further discussed under understandability.

Understandability revealed structural shortcomings in health information conveyance. While all models performed excellently on clarity of material purpose and content focus (means ≥ 0.96), performance on plain language use and appropriate definition of medical terms was far less satisfactory. Even DeepSeek-V4 adequately explained medical terms in only 58% of responses, with Qwen3.8-Max-Preview as low as 34%. This carries profound implications given that many PLWHA in China come from rural areas or have lower education levels (45); unexplained jargon such as “CD4+ T lymphocytes” or “antiretroviral therapy” negates practical utility even when information is accurate and complete (50). Current models exhibit a contradiction between completeness and understandability: information is comprehensive but potentially incomprehensible. This asymmetry may exacerbate health inequities, constituting a covert reproduction of the digital health divide (40). DeepSeek-V4 scored significantly higher than HuatuoGPT-II (*P* = 0.001) and Yuanbao-Hy3 (*P* = 0.004) on composite understandability, primarily due to superior plain language performance (M = 0.740 vs. 0.380 for Yuanbao-Hy3), indicating substantive differences in “knowledge downscaling.” HuatuoGPT-II's lag on information chunking and visual cue effectiveness further confirms that medical-domain-specific models, as exemplified by HuatuoGPT-II, may be inadvertently optimized toward academic or clinically codified expressive paradigms that are inherently less aligned with the principles of layperson-oriented health communication.

Actionability, the bridge from understanding to action, was universally low (maximum 1.70 out of 3 for DeepSeek-V4), indicating a systemic deficiency in translating knowledge into executable behavioral guidance. Together with insufficient term explanation, this points to a core contradiction: models excel at “informing” but are poor at “guiding action.” At the item level, models performed evenly on identification of actions (*P* = 0.273), suggesting universal basic capacity. However, differences were extremely significant for addressing the user directly (Item 9; *P* < 0.001): even DeepSeek-V4 used second-person address in only 40% of responses, with Doubao 2.1 as low as 4%, making it difficult for readers to perceive personal relevance. On manageability of steps (Item 10), DeepSeek-V4 (48%) and Yuanbao-Hy3 (46%) performed relatively best, while HuatuoGPT-II scored only 8%, significantly lagging behind. Taken together, these findings suggest a potential limitation of the medical-domain-specific model examined here: despite its likely rich exposure to clinical pathway corpora, it may be comparatively less effective at condensing specialized medical knowledge into step-by-step, lay-executable plans. This interpretation, however, remains tentative and should be tested in future studies that directly compare the fine-tuning objectives and output generation strategies of different model types. Insufficient actionability means outputs remain at “knowledge transfer” level without effectively guiding behavior (46), consistent with Deng et al. (33) on ChatGPT for patellar tendinopathy. For PLWHA with lower health literacy, absence of second-person action guidance may leave them at a loss at critical decision points. Future alignment training should strengthen patient-centred, action-oriented paradigms, upgrading from “stating facts” to “guiding action.”

The uniform application of Caring and Actionability scoring across all questions may also shape how the results are interpreted. Because the scoring criteria do not vary by question type, purely factual questions are assessed on the same scale for empathic expression and action guidance as psychologically sensitive ones. This may lower overall scores on these two dimensions, especially when the question itself carries little emotional burden. In HIV/AIDS counseling, however, anti-stigma communication and actionable guidance are not optional additions. Stigma remains a central barrier to testing, treatment, and open discussion, while actionable guidance determines whether users can translate information into testing, prevention, or care-seeking behavior. If models show Caring and Actionability levels on sensitive questions that are similar to those on factual questions, such static and non-adaptive scoring functions as a stress test, exposing generalization deficits in socio-emotional reasoning and action-oriented translation. In this sense, the lower scores produced by uniform scoring are not merely an artifact of strict criteria. They are more likely to reveal that the models fail to recognize contextual differences and fail to adjust to the core demands of anti-stigma communication and action support in HIV/AIDS counseling.

Although variable-centred analyses reveal independent performance, they presuppose sample homogeneity and fail to capture combinatorial quality patterns. The four profiles revealed distinct quality typologies. Profile 3, accounting for half of all responses, confirmed the dominance of technically accurate but humanistically moderate outputs. In contrast, Profile 4, characterized by perfect completeness and the highest actionability, suggested that some responses achieve a pragmatic balance by prioritizing actionable guidance and comprehensive coverage, even at the expense of a slight reduction in factual precision and empathetic tone compared to Profile 3. Notably, the Moderate-Balanced Profile (Profile 2) had the highest caring score, indicating that empathetic engagement was not necessarily coupled with technical excellence or completeness, and that different models may excel in different facets of quality. For HIV/AIDS, these humanistically balanced patterns may hold greater practical value than purely technical optimality, as information adoption depends on emotional support and feasibility (35, 47). Understandability showed no significant profile differences (*P* = 0.194) and occupied the most peripheral network position (strength = 0.151), indicating a ceiling effect: current LLMs have internalized readability regularities, shifting understandability from bottleneck to foundational capability. Future evaluation frameworks may need to reduce its weight and increase measurement of personalization, cultural adaptability, and behavioral activation. Partial correlation network analysis revealed that completeness occupied the topologically most central position (strength = 0.813, closeness = 1.000), maintaining positive conditional associations with accuracy and actionability after controlling for other dimensions. From a network topology perspective, high expected influence indicates that a node possesses numerous and relatively strong conditional association edges with other dimensions; its statistical fluctuation is theoretically associated with broader patterns of network co-variation (28, 29). Actionability shared the highest betweenness centrality (0.167), serving as a statistical bridge connecting the technical and humanistic clusters within the conditional dependence structure. Betweenness centrality, as a topological indicator, quantifies the extent to which a node lies on the shortest pathways between other node pairs, reflecting its role in information flow efficiency and cross-community structural integration within the network (30). However, it is critical to recognize that these centrality indices are descriptive topological features rather than evidence of causal priority or directional intervention effects (26, 27). Cross-sectional network models cannot distinguish whether completeness drives co-variation among other dimensions or merely exhibits high connectivity because it shares common latent confounders with multiple dimensions. Accordingly, the centrality of completeness is strictly defined herein as a statistical hub within the co-variation structure of the evaluation framework, not as a validated causal lever. Any inference that prioritizing improvements in completeness and actionability would necessarily yield cascading effects across other dimensions should be regarded as a working hypothesis warranting longitudinal or experimental validation, rather than a conclusion supported by the present cross-sectional analysis. A weak negative partial correlation between accuracy and actionability (*r* = −0.083) suggested a potential trade-off between technical precision and practical guidance at the statistical level. However, this cross-sectional association does not establish a causal crowding-out effect. Alternative interpretations, including latent confounding (e.g., response length, domain complexity) or semantic competition during output generation, remain plausible. The observed pattern is nevertheless consistent with cross-linguistic evidence suggesting similar tensions between comprehensiveness and actionability in health information delivery (33, 49). Chi-square tests revealed highly significant model differences in profile distribution (χ^2^ = 124.99, *P* < 0.001) but no domain differences (*P* = 0.625), indicating heterogeneity stems from model architecture rather than question content. Thus, Yuanbao-Hy3 may be preferable to DeepSeek-V4 for psychological support despite lower technical scores. Domain-specific fine-tuning may have less impact than enhancing base model capabilities. However, further investigation is warranted, as our evaluation assessed textual actionability rather than actual patient behaviors, comprehension, or downstream clinical responses.

If replicated in future confirmatory studies with larger and more diverse question banks, our findings would offer preliminary guidance for multiple stakeholders. From a policy perspective, our observation that the single medical-domain-specific model examined did not outperform the four general-purpose models on most dimensions particularly in public-facing health education contexts suggests that the assumption of inherent superiority of domain-specialized systems warrants empirical scrutiny rather than uncritical acceptance. However, because we evaluated only one such model, this observation should not be generalized to all medical-domain-specific LLMs. For model developers, our findings point to specific areas for potential improvement, such as empathetic expression corpora, de-stigmatizing language norms, and action-oriented feedback signals in reinforcement learning. Though these recommendations derive from output-level evaluations and would benefit from direct experimental validation through controlled fine-tuning studies. Clinicians should continue to position AI as supplementary to, rather than a replacement for, professional medical guidance, recognizing that current LLMs outputs exhibit systematic deficits in caring and actionability that limit their readiness for independent patient-facing use. Users must rely on medical institutions for clinical decisions to avoid misjudgements from over-reliance on AI.

Four limitations delimit the present findings. First, the subjective rating scales may be vulnerable to ceiling or floor effects, as accuracy and completeness approached the maximum while caring remained generally low, potentially compressing genuine between-dimension differences and attenuating measurement sensitivity. Second, each model output was assessed after a single query without repeated sampling, so stochastic variability for identical inputs remains unobserved, constraining reproducibility estimation and potentially introducing bias, and although three-rater consensus controlled for human measurement error, model-level stochastic uncertainty remains unquantified. Third, the modest question bank (*n* = 50, yielding 250 observations) was adequately powered for moderate-to-large effects in mixed-effects models but limits LPA class stability and the precision of network centrality estimates, and one profile contained only nine responses (3.6%), below the conventional 5% threshold, so the profile typology and network topology should be regarded as provisional and hypothesis-generating pending validation in larger, independent samples. Fourth, the partial correlation network is exploratory and cross-sectional, and its centrality indices describe conditional dependence patterns in the current data rather than causal priority or intervention targets (26, 27), while the single-time-point design also precludes assessment of temporal stability and makes it difficult to characterize version-to-version or timeliness changes, thereby limiting causal inference and longitudinal extrapolation. Addressing these limitations, future research should proceed along four lines. First, scoring granularity should be refined and real-user comprehension and behavioral outcomes incorporated to mitigate ceiling or floor effects and enhance measurement sensitivity. Second, repeated runs of 5–10 per prompt should become standard so that model-level stochastic uncertainty can be quantified alongside human measurement error and output reproducibility assessed. Third, larger, independently sampled and linguistically diverse question banks, together with multi-turn dialogue protocols, are needed to validate LPA class solutions, the profile typology and network centrality estimates. Fourth, longitudinal or experimental designs should test directionality, causal priority and temporal stability among dimensions and track version-to-version change, thereby moving evaluation of Chinese LLMs in HIV/AIDS communication from exploratory mapping toward confirmatory, person-centred and clinically actionable assessment.

## 5 Conclusion

This study systematically evaluated the multidimensional performance of five Chinese LLMs in HIV/AIDS health education Q&A across five dimensions: accuracy, completeness, understandability, actionability, and caring. Within the constraints of our 50-question framework and limited to the specific models evaluated, the four general-purpose models attained high levels of factual accuracy and information completeness, indicative of a foundational capacity that may support their use as public HIV/AIDS knowledge provision channels; however, they exhibited consistent structural deficiencies in caring expression, plain language use, and action guidance. The single medical-domain-specific model included in this study did not demonstrate anticipated advantages in public-facing health communication scenarios, although this finding is specific to the single model examined and cannot be extrapolated to other medical-domain-specific systems without further evidence. Our LPA and network analyses—interpreted with appropriate caution given their exploratory nature—revealed that heterogeneity in response quality is driven primarily by model-specific characteristics rather than question content, and that completeness and actionability occupy topologically central positions within the conditional dependence structure, thereby offering a set of testable hypotheses that warrant confirmation through future confirmatory or experimental designs. It bears emphasizing that these findings are inherently delimited by the specific set of models, the fixed question bank, the single-turn query format, and the Mandarin-only linguistic scope; accordingly, they should be construed as hypothesis-generating evidence that maps the current performance landscape of a selected group of Chinese LLMs, rather than as definitive or broadly generalizable conclusions. Future investigations should give priority to external validation using larger, independently sampled question banks, repeated query protocols, multi-turn dialogue assessments, and real-user behavioral outcome measures, so as to determine whether the patterns observed here reflect reproducible properties of Chinese LLMs in HIV/AIDS health communication or are contingent upon our specific evaluation design.

## Data Availability

The original contributions presented in the study are included in the article/Supplementary material, further inquiries can be directed to the corresponding authors.
