# Supplementary material for: Evaluating Chinese large language models for HIV/AIDS health information: a multidimensional comparative study of response quality
Source: Front Public Health. 2026 Oct 6;14:1966522. doi: 10.3389/fpubh.2026.1966522 (PMC13680460; doi:10.3389/fpubh.2026.1966522)
Supplement: Supplementary file 1 [file Data_Sheet_1.docx]

Supplement to: Evaluating Chinese Large Language Models for HIV/AIDS Health Information: A Multidimensional Comparative Study of Response Quality

**Contents**

**Table S1.** Fifty questions about HIV

**Table S2.** Five‑Point Likert Scale for Evaluating the Accuracy of LLM Response

**Table S3.** Five‑Point Likert Scale for Evaluating the Completeness of LLM Responses

**Table S4.**Five‑Point Likert Scale for Evaluating the Caring of LLM Responses

**Table S5.** PEMAT Dimensions for Evaluating the Understandability of LLM Responses

**Table S6.** PEMAT Dimensions for Evaluating the Actionability of LLM Responses

**Table S7.** Inter-rater Reliability Coefficients for Each Model and Dimension about Accuracy

**Table S8.** Inter-rater Reliability Coefficients for Each Model and Dimension about Caring

**Table S9.** Inter-rater Reliability Coefficients for Each Model and Dimension about Completeness

**Table S10.** Inter-rater Reliability Coefficients for Each Model and Dimension about Understandability

**Table S11.** Inter-rater Reliability Coefficients for Each Model and Dimension about Actionability

**Table S12.** Pairwise Comparisons for Accuracy Using Cumulative Link Mixed Model (CLMM)

**Table S13.** Pairwise Comparisons for Accuracy Using Wilcoxon Signed-Rank Test

**Table S14.** Pairwise Comparisons for Caring Using Cumulative Link Mixed Model (CLMM)

**Table S15.** Pairwise Comparisons for Caring Using Wilcoxon Signed-Rank Test

**Table S16.** Pairwise Comparisons for Completeness Using Cumulative Link Mixed Model (CLMM)

**Table S17.** Pairwise Comparisons for Completeness Using Wilcoxon Signed-Rank Test

**Table S18.** Concordance of significance decisions between cumulative link mixed model and Wilcoxon signed-rank test under FDR correction.

**Table S19.**Pairwise *P*-values under Holm correction for all three dimensions.

**Table S20.**Pairwise *P*-values under Bonferroni correction for all three dimensions.

**Table S21.** Overall Differences and Significant Pairwise Comparisons for Understandability

**Table S22.** Pairwise comparisons for Understandability items and total score under Holm and Bonferroni corrections.

**Table S23.** Overall Differences and Significant Pairwise Comparisons for Actionability

**Table S24.** Pairwise comparisons for Actionability items and total score under Holm and Bonferroni corrections.

**Table S25.** Between-group comparisons of the four latent profiles across quality dimensions.

**Table S26** Regularized Partial Correlation Matrix Among Five AI Response Quality Dimensions Estimated via Graphical Lasso

**Table S27.**Robustness Checks for Question-Level Dependency in LPA and Network Analyses.

**Supplementary Tables**

**Table S1.** Fifty questions about HIV

| No. | Question |
| --- | --- |
| 1 | What is the difference between AIDS and HIV? |
| 2 | Which system of the human body does HIV primarily attack, and how does it cause immune function decline? |
| 3 | What are the routes of HIV transmission, and which bodily fluids are infectious? |
| 4 | How long can HIV survive outside the body, and what are the survival conditions in everyday environments? |
| 5 | After HIV infection, what are the usual stages of disease progression, and what are the characteristics of each stage? |
| 6 | What is the "window period" for HIV? What test results might appear during the window period? |
| 7 | Does HIV have specific clinical symptoms? What are the manifestations during the acute phase and the latent phase? |
| 8 | What are opportunistic infections, and what are the common HIV‑related opportunistic infections? |
| 9 | Why do men who have sex with men (MSM) have a higher risk of HIV infection? |
| 10 | Can two apparently healthy people contract HIV through unprotected sex? Why? |
| 11 | What is the "ABC principle" for preventing sexual transmission of HIV? |
| 12 | Can consistent and correct condom use during every sexual encounter provide 100% protection against HIV? What are the reasons for failure? |
| 13 | Is it safer to use two condoms at the same time? Does it provide better protection against HIV? |
| 14 | What is pre‑exposure prophylaxis (PrEP)? Who is it suitable for, and what are the dosing options? |
| 15 | What is post‑exposure prophylaxis (PEP)? What is the time window for taking it, and what is the course duration? |
| 16 | After high‑risk sexual behavior, what emergency measures should be taken in addition to PEP? |
| 17 | How can people who inject drugs effectively prevent HIV infection? What are the alternatives or harm‑reduction approaches? |
| 18 | Does having a sexually transmitted infection (STI) increase the risk of HIV infection? Why? |
| 19 | Can oral sex transmit HIV? Under what circumstances does oral sex pose a risk of infection? |
| 20 | Can mosquito bites transmit HIV? Can daily contact (such as hugging, sharing meals, or using public toilets) transmit HIV? |
| 21 | What is the only way to confirm HIV infection, and why can't symptoms be relied upon for diagnosis? |
| 22 | What are the commonly used HIV testing methods, and what are their respective window periods? |
| 23 | After high‑risk behavior, during which weeks is it most accurate to get HIV nucleic acid testing, antigen testing, and antibody testing, respectively? |
| 24 | Where in China can one receive free, confidential HIV testing? What does VCT clinic mean? |
| 25 | If an HIV test result is negative, does it mean there is definitely no infection? What if it is still within the window period? |
| 26 | If an HIV test result is positive (reactive), what should be done next? Is a confirmatory test needed? |
| 27 | Does taking common medications (such as cold medicines or antibiotics) or having a fever due to illness affect HIV antibody test results? |
| 28 | Are HIV self‑test kits purchased online accurate? What should one do after a positive self‑test result? |
| 29 | Is real‑name registration required for HIV testing in China? Will the test results be disclosed to family members or employers? |
| 30 | Why is it recommended that people living with HIV undergo tuberculosis screening at least once a year? |
| 31 | Can AIDS be completely cured at present? What is a "functional cure"? |
| 32 | Since HIV cannot be cured, why is it still necessary to adhere to antiretroviral therapy? What are the benefits of treatment? |
| 33 | What is the basic principle of antiretroviral therapy (ART / "cocktail therapy"), and what are the main classes of drugs used? |
| 34 | Does China provide free antiretroviral drugs to people living with HIV? What are the requirements? |
| 35 | During HIV treatment, how can one determine whether the medication is effective? |
| 36 | What does "U=U" mean? After achieving viral suppression, how high is the risk of sexual transmission of HIV? |
| 37 | What are the consequences of missing doses during HIV treatment, and why is it necessary to maintain adherence above 95%? |
| 38 | While on antiretroviral therapy, if other illnesses occur (such as a cold or hypertension), can one add other medications on their own? |
| 39 | What are the common side effects of HIV antiretroviral drugs, and how should serious adverse reactions be handled? |
| 40 | Can people living with HIV receive traditional Chinese medicine (TCM) treatment? Can doctors mandate the use of TCM? |
| 41 | After HIV infection, can one still attend school, work, and get married normally? |
| 42 | How can people living with HIV maintain a reasonable nutritional balance, and what are the dietary precautions? |
| 43 | Can people living with HIV exercise? What are the recommended frequency and intensity of exercise? |
| 44 | Can people living with HIV smoke or drink alcohol? What are the impacts on health? |
| 45 | How can one adjust their mindset after HIV infection, overcome fear and anxiety, and what psychological support options are available? |
| 46 | If there is a person living with HIV in the household, what precautions should family members take in daily life? Is it necessary to separate utensils? |
| 47 | Can people living with HIV give birth to healthy babies? What does prevention of mother‑to‑child transmission (PMTCT) include? |
| 48 | After HIV infection, can one still have a normal sex life? What should be noted? |
| 49 | What is "AIDS phobia"? Can it cause real physical symptoms, and how should it be addressed? |
| 50 | Is unprotected sex between two people living with HIV safe? Why? |

**Table S2.** Five‑Point Likert Scale for Evaluating the Accuracy of LLM Responses

| Score | Definition |
| --- | --- |
| 5 | Almost entirely correct / completely correct |
| 4 | More correct than incorrect |
| 3 | Roughly equal correct and incorrect |
| 2 | More incorrect than correct |
| 1 | Completely incorrect |

**Table S3.** Five‑Point Likert Scale for Evaluating the Completeness of LLM Responses

| Score | Definition |
| --- | --- |
| 5 | Comprehensive (addresses all aspects of the question and provides useful additional information or context beyond expectations) |
| 4 | Sufficient (addresses all aspects of the question and provides a small amount of useful supplemental information) |
| 3 | Complete (addresses all aspects of the question, providing only the minimum information to consider) |
| 2 | Incomplete (addresses only some aspects of the question, with important parts missing, incomplete, or unhelpful) |
| 1 | Completely incomplete (does not address any aspect of the question) |

**Table S4.**Five‑Point Likert Scale for Evaluating the Caring of LLM Responses

| Score | Definition |
| --- | --- |
| 5 | Deep empathy and active destigmatisation |
| 4 | Mild empathy and explicit anti‑discrimination |
| 3 | Factually neutral / zero emotional tone |
| 2 | Implicit bias / mild stigmatisation |
| 1 | Overt discrimination and highly malicious |

**Table S5.** PEMAT Dimensions for Evaluating the Understandability of LLM Responses

| Item | Category | Scoring | Explanation |
| --- | --- | --- | --- |
| 1 | Purpose evident | Disagree = 0; Agree = 1 | The material must clearly inform the reader of its core purpose through the title or opening content. If it only uses vague statements such as “This material helps you understand health knowledge” without specifying the concrete topic, choose “Disagree”; if it accurately describes the core subject, choose “Agree”. |
| 2 | Content focus | Disagree = 0; Agree = 1 | The material must stay tightly focused on its core purpose without extraneous information. Only essential content should be retained, avoiding distracting details (e.g., device principles, disease history) that may cause information overload. |
| 3 | Common language | Disagree = 0; Agree = 1 | The material should use everyday language that is easy for the public to understand, avoiding jargon. If abbreviations or acronyms appear, they must be fully spelled out and explained at first use; if there are too many abbreviations (even if explained) that hinder reading, choose “Disagree”. Common medical abbreviations such as “CT (computed tomography)” or “B‑ultrasound (B‑mode ultrasonography)” do not require rejection solely for their use. |
| 4 | Medical terms defined | Disagree = 0; Agree = 1 | The material should avoid using medical terms unless it is intended to “educate the reader about the terms”, in which case they must be accompanied by a plain‑language explanation. Providing explanations only via hyperlinks (e.g., clicking the term to jump to an explanation page) is not sufficient, as readers may not click or may have difficulty operating the link; hyperlinks can be supplementary but must be combined with a direct explanation. |
| 5 | Information chunking | Disagree = 0; Agree = 1; Not applicable for very short materials (≤2 sentences) | The material should break information into short “chunks” to avoid long blocks of text that cause information overload. Even long lists (e.g., “10 health tips”) should be divided into sub‑lists of 2–3 items to facilitate comprehension and memory. |
| 6 | Logical organization and clear flow | Disagree = 0; Agree = 1; Not applicable for very short materials (≤2 sentences) | Information should be presented in an order that is meaningful to the reader, not necessarily following a chronological or “background‑core” structure, but with a smooth flow. Key messages and important points should be placed at the beginning of paragraphs or lists (readers tend to pay more attention to opening content), with details progressively elaborated. |
| 7 | Visual cue effectiveness | Disagree = 0; Agree = 1 | The material should use visual cues (e.g., arrows, boxes, bullet points, boldface, large fonts, colour highlights) to emphasise key information, but such cues should only be used for “core points”. Excessive use (e.g., bolding entire paragraphs, using bullet points for every sentence) makes it difficult for the reader to distinguish priorities, in which case choose “Disagree”. |

**Table S6.** PEMAT Dimensions for Evaluating the Actionability of LLM Responses

| Item | Category | Scoring | Explanation |
| --- | --- | --- | --- |
| 8 | Action identified | Disagree = 0; Agree = 1 | The material must clearly specify at least one concrete action that the reader can take, avoiding statements that merely present facts or data without behavioural guidance. If no actionable behaviour is mentioned, choose “Disagree”. |
| 9 | Direct address | Disagree = 0; Agree = 1 | When describing actions, the material should directly address the reader using second‑person pronouns (e.g., “you”, “your”) so that the reader clearly understands “this is what I need to do”. If no action is described at all, choose “Disagree”; if an action is described but uses third‑person or impersonal phrasing, also choose “Disagree”. |
| 10 | Manageable steps | Disagree = 0; Agree = 1 | The material should break down actions into “operational, easy‑to‑execute concrete steps”, especially for complex behaviours such as “increase physical activity” or “control diet”. It should clearly specify “step one, step two”. Avoid using vague terms like “often”, “moderately”, or “sufficiently”, and do not present only a goal (e.g., “lose weight”) without providing the means to achieve it. |

**Table S7.** Inter-rater Reliability Coefficients for Each Model and Dimension about Accuracy

| **Indicator** | **DeepSeek-V4** | **Doubao2.1** | **HuatuoGPT-II** | **Qwen3.8-Max-Preview** | **Yuanbao-Hy3** |
| --- | --- | --- | --- | --- | --- |
| Cohen's κ (Rater1-Rater2) | 0.875 | 0.868 | 0.852 | 0.882 | 0.797 |
| Cohen's κ (Rater1-Rater3) | 0.789 | 0.649 | 0.825 | 0.756 | 0.77 |
| Cohen's κ (Rater2-Rater3) | 0.789 | 0.742 | 0.939 | 0.87 | 0.814 |
| Exact Agreement (%) | 90 | 84 | 78 | 86 | 84 |
| Gwet's AC1 | 0.924 | 0.871 | 0.816 | 0.876 | 0.864 |
| Krippendorff's α (ordinal) | 0.747 | 0.77 | 0.897 | 0.859 | 0.814 |
| N | 50 | 50 | 50 | 50 | 50 |
| PABAK | 0.867 | 0.787 | 0.707 | 0.813 | 0.787 |
| Raters | 3 | 3 | 3 | 3 | 3 |
| Weighted Fleiss' κ | 0.814 | 0.756 | 0.875 | 0.834 | 0.793 |

**Table S8.** Inter-rater Reliability Coefficients for Each Model and Dimension about Caring

| Indicator | DeepSeek-V4 | Doubao2.1 | HuatuoGPT-II | Qwen3.8-Max-Preview | Yuanbao-Hy3 |
| --- | --- | --- | --- | --- | --- |
| Cohen's κ (Rater1-Rater2) | 0.956 | 0.929 | 0.964 | 0.911 | 0.969 |
| Cohen's κ (Rater1-Rater3) | 0.941 | 0.925 | 0.961 | 0.943 | 0.954 |
| Cohen's κ (Rater2-Rater3) | 0.894 | 0.943 | 0.919 | 0.898 | 0.922 |
| Exact Agreement (%) | 90 | 92 | 96 | 88 | 90 |
| Gwet's AC1 | 0.908 | 0.934 | 0.97 | 0.892 | 0.905 |
| Krippendorff's α (ordinal) | 0.946 | 0.964 | 0.948 | 0.961 | 0.955 |
| N | 50 | 50 | 50 | 50 | 50 |
| PABAK | 0.867 | 0.893 | 0.947 | 0.84 | 0.867 |
| Raters | 3 | 3 | 3 | 3 | 3 |
| Weighted Fleiss' κ | 0.93 | 0.932 | 0.948 | 0.918 | 0.948 |

**Table S9.** Inter-rater Reliability Coefficients for Each Model and Dimension about Completeness

| Indicator | DeepSeek-V4 | Doubao2.1 | HuatuoGPT-II | Qwen3.8-Max-Preview | Yuanbao-Hy3 |
| --- | --- | --- | --- | --- | --- |
| Cohen's κ (Rater1-Rater2) | 0.746 | 0.728 | 0.868 | 0.925 | 1 |
| Cohen's κ (Rater1-Rater3) | 0.841 | 0.878 | 0.85 | 0.864 | 0.79 |
| Cohen's κ (Rater2-Rater3) | 0.935 | 0.878 | 0.896 | 0.928 | 0.79 |
| Exact Agreement (%) | 94 | 96 | 80 | 90 | 98 |
| Gwet's AC1 | 0.956 | 0.968 | 0.834 | 0.928 | 0.985 |
| Krippendorff's α (ordinal) | 0.864 | 0.833 | 0.888 | 0.947 | 0.869 |
| N | 50 | 50 | 50 | 50 | 50 |
| PABAK | 0.92 | 0.947 | 0.733 | 0.867 | 0.973 |
| Raters | 3 | 3 | 3 | 3 | 3 |
| Weighted Fleiss' κ | 0.849 | 0.832 | 0.872 | 0.905 | 0.868 |

**Table S10.** Inter-rater Reliability Coefficients for Each Model and Dimension about Understandability

| Indicator | DeepSeek-V4 | Doubao2.1 | HuatuoGPT-II | Qwen3.8-Max-Preview | Yuanbao-Hy3 |
| --- | --- | --- | --- | --- | --- |
| Agreement Level (based on AC1) | Excellent | Excellent | Excellent | Excellent | Excellent |
| Ceiling/Floor Effect | Ceiling Effect | Normal | Normal | Normal | Normal |
| Fleiss' Kappa | 0.821 | 0.873 | 0.914 | 0.955 | 0.98 |
| Gwet's AC1 | 0.966 | 0.956 | 0.958 | 0.985 | 0.992 |
| Kappa Paradox Risk | Medium Risk | Low Risk | Low Risk | Low Risk | Low Risk |
| Kappa-AC1 Difference | 0.145 | 0.083 | 0.044 | 0.03 | 0.012 |
| Krippendorff's Alpha | 0.821 | 0.873 | 0.921 | 0.955 | 0.98 |
| Mean Pairwise Cohen's Kappa | 0.821 | 0.873 | 0.92 | 0.955 | 0.98 |
| Number of Items | 350 | 350 | 350 | 350 | 350 |
| Number of Raters | 3 | 3 | 3 | 3 | 3 |
| PABAK | 0.943 | 0.935 | 0.945 | 0.977 | 0.989 |
| Percent Agreement (PA) | 0.957 | 0.951 | 0.96 | 0.983 | 0.991 |
| Prevalence of Category 1 | 0.912 | 0.85 | 0.796 | 0.851 | 0.823 |
| Recommended Primary Index | Gwet's AC1 | Fleiss' Kappa / AC1 both acceptable | Fleiss' Kappa / AC1 both acceptable | Gwet's AC1 | Fleiss' Kappa / AC1 both acceptable |

**Table S11.** Inter-rater Reliability Coefficients for Each Model and Dimension about Actionability

| Indicator | DeepSeek-V4 | Doubao2.1 | HuatuoGPT-II | Qwen3.8-Max-Preview | Yuanbao-Hy3 |
| --- | --- | --- | --- | --- | --- |
| Agreement Level (based on AC1) | Good | Excellent | Excellent | Excellent | Good |
| Ceiling/Floor Effect | Normal | Normal | Normal | Normal | Normal |
| Fleiss' Kappa | 0.822 | 0.906 | 0.952 | 0.901 | 0.89 |
| Gwet's AC1 | 0.839 | 0.916 | 0.959 | 0.903 | 0.896 |
| Kappa Paradox Risk | Low Risk | Low Risk | Low Risk | Low Risk | Low Risk |
| Kappa-AC1 Difference | 0.017 | 0.01 | 0.008 | 0.002 | 0.006 |
| Krippendorff's Alpha | 0.822 | 0.906 | 0.952 | 0.901 | 0.89 |
| Mean Pairwise Cohen's Kappa | 0.822 | 0.906 | 0.951 | 0.901 | 0.89 |
| Number of Items | 150 | 150 | 150 | 150 | 150 |
| Number of Raters | 3 | 3 | 3 | 3 | 3 |
| PABAK | 0.831 | 0.911 | 0.956 | 0.902 | 0.893 |
| Percent Agreement (PA) | 0.873 | 0.933 | 0.967 | 0.927 | 0.92 |
| Prevalence of Category 1 | 0.613 | 0.382 | 0.356 | 0.451 | 0.58 |
| Recommended Primary Index | Fleiss' Kappa / AC1 both acceptable | Fleiss' Kappa / AC1 both acceptable | Fleiss' Kappa / AC1 both acceptable | Fleiss' Kappa / AC1 both acceptable | Fleiss' Kappa / AC1 both acceptable |

**Table S12.** Pairwise Comparisons for Accuracy Using Cumulative Link Mixed Model (CLMM)

| Comparison | CLMM beta (SE) | CLMM OR (95% CI) | CLMM z | CLMM *P*-FDR |
| --- | --- | --- | --- | --- |
| DeepSeek-V4 vs HuatuoGPT-II | 2.458 (0.488) | 11.68 (4.47-30.48) | 5.03 | <0.001 |
| Doubao2.1 vs HuatuoGPT-II | 1.726 (0.453) | 5.62 (2.31-13.63) | 3.81 | <0.001 |
| HuatuoGPT-II vs Qwen3.8-Max-Preview | -1.887 (0.459) | 0.15 (0.06-0.38) | -4.12 | <0.001 |
| HuatuoGPT-II vs Yuanbao-Hy3 | -2.047 (0.467) | 0.13 (0.05-0.33) | -4.39 | <0.001 |
| DeepSeek-V4 vs Doubao2.1 | 0.733 (0.455) | 2.08 (0.86-5.06) | 1.61 | 0.215 |
| DeepSeek-V4 vs Qwen3.8-Max-Preview | 0.571 (0.457) | 1.77 (0.72-4.32) | 1.25 | 0.353 |
| DeepSeek-V4 vs Yuanbao-Hy3 | 0.411 (0.459) | 1.51 (0.61-3.69) | 0.90 | 0.530 |
| Doubao2.1 vs Qwen3.8-Max-Preview | -0.161 (0.436) | 0.85 (0.36-1.99) | -0.37 | 0.717 |
| Doubao2.1 vs Yuanbao-Hy3 | -0.322 (0.439) | 0.72 (0.31-1.71) | -0.73 | 0.580 |
| Qwen3.8-Max-Preview vs Yuanbao-Hy3 | -0.160 (0.442) | 0.85 (0.36-2.01) | -0.36 | 0.717 |

Note. CLMM = cumulative link mixed model with question ID as random intercept; OR = odds ratio; CI = confidence interval; FDR = Benjamini–Hochberg false discovery rate. Beta > 0 indicates that Model 1 scored higher than Model 2. Bold values indicate *P*-FDR < 0.05. Overall test: likelihood-ratio χ² = 34.83, df = 4, *P* < 0.001; proportional-odds assumption *P* = 0.396 (met).

**Table S13.** Pairwise Comparisons for Accuracy Using Wilcoxon Signed-Rank Test

| Comparison | Wilcoxon Z | Wilcoxon r | Wilcoxon *P*-FDR |
| --- | --- | --- | --- |
| DeepSeek-V4 vs HuatuoGPT-II | 4.21 | 0.59 | <0.001 |
| Doubao2.1 vs HuatuoGPT-II | 3.53 | 0.50 | 0.001 |
| HuatuoGPT-II vs Qwen3.8-Max-Preview | 3.98 | 0.56 | <0.001 |
| HuatuoGPT-II vs Yuanbao-Hy3 | 3.77 | 0.53 | <0.001 |
| DeepSeek-V4 vs Doubao2.1 | 1.63 | 0.23 | 0.205 |
| DeepSeek-V4 vs Qwen3.8-Max-Preview | 1.41 | 0.20 | 0.262 |
| DeepSeek-V4 vs Yuanbao-Hy3 | 0.89 | 0.13 | 0.464 |
| Doubao2.1 vs Qwen3.8-Max-Preview | 0.43 | 0.06 | 0.683 |
| Doubao2.1 vs Yuanbao-Hy3 | 0.89 | 0.13 | 0.464 |
| Qwen3.8-Max-Preview vs Yuanbao-Hy3 | 0.41 | 0.06 | 0.683 |

Note. Wilcoxon signed-rank test with Benjamini–Hochberg FDR correction. Z = Wilcoxon Z statistic; r = effect size (Z/√N, where N = 50); r of 0.1, 0.3, and 0.5 correspond to small, medium, and large effects, respectively. Bold values indicate P-FDR < 0.05. Overall test: permutation-based Friedman χ² = 18.96, *P* = 0.0001, Kendall’s W = 0.095.

Table S14. Pairwise Comparisons for Caring Using Cumulative Link Mixed Model (CLMM)

| Comparison | CLMM beta (SE) | CLMM OR (95% CI) | CLMM z | CLMM *P*-FDR |
| --- | --- | --- | --- | --- |
| DeepSeek-V4 vs HuatuoGPT-II | 2.699 (0.609) | 14.86 (4.49–49.17) | 4.43 | <0.001 |
| HuatuoGPT-II vs Yuanbao-Hy3 | −2.860 (0.614) | 0.06 (0.02–0.17) | −4.66 | <0.001 |
| HuatuoGPT-II vs Qwen3.8-Max-Preview | −2.271 (0.609) | 0.10 (0.03–0.35) | −3.73 | <0.001 |
| DeepSeek-V4 vs Doubao2.1 | 1.353 (0.505) | 3.87 (1.44–10.42) | 2.68 | 0.015 |
| Doubao2.1 vs Yuanbao-Hy3 | −1.514 (0.508) | 0.22 (0.08–0.60) | −2.98 | 0.007 |
| Doubao2.1 vs HuatuoGPT-II | 1.345 (0.605) | 3.84 (1.17–12.58) | 2.22 | 0.044 |
| Doubao2.1 vs Qwen3.8-Max-Preview | −0.925 (0.512) | 0.40 (0.15–1.08) | −1.81 | 0.101 |
| Qwen3.8-Max-Preview vs Yuanbao-Hy3 | −0.589 (0.472) | 0.55 (0.22–1.39) | −1.25 | 0.265 |
| DeepSeek-V4 vs Qwen3.8-Max-Preview | 0.428 (0.471) | 1.53 (0.61–3.86) | 0.91 | 0.405 |
| DeepSeek-V4 vs Yuanbao-Hy3 | −0.161 (0.456) | 0.85 (0.35–2.07) | −0.35 | 0.724 |

Note. CLMM = cumulative link mixed model with question ID as random intercept; OR = odds ratio; CI = confidence interval; FDR = Benjamini–Hochberg false discovery rate. Beta > 0 indicates that Model 1 scored higher than Model 2. Bold values indicate *P*-FDR < 0.05. Overall test: likelihood-ratio χ² = 35.20, df = 4, *P* < 0.001; proportional-odds assumption *P* = 0.796 (met).

**Table S15.** Pairwise Comparisons for Caring Using Wilcoxon Signed-Rank Test

| Comparison | Wilcoxon Z | Wilcoxon r | Wilcoxon *P*-FDR |
| --- | --- | --- | --- |
| DeepSeek-V4 vs Doubao2.1 | 2.98 | 0.42 | 0.006 |
| DeepSeek-V4 vs HuatuoGPT-II | 3.70 | 0.52 | 0.001 |
| DeepSeek-V4 vs Qwen3.8-Max-Preview | 0.86 | 0.12 | 0.433 |
| DeepSeek-V4 vs Yuanbao-Hy3 | 0.17 | 0.02 | 0.864 |
| Doubao2.1 vs HuatuoGPT-II | 2.24 | 0.32 | 0.042 |
| Doubao2.1 vs Qwen3.8-Max-Preview | 1.98 | 0.28 | 0.069 |
| Doubao2.1 vs Yuanbao-Hy3 | 3.01 | 0.43 | 0.006 |
| HuatuoGPT-II vs Qwen3.8-Max-Preview | 3.39 | 0.48 | 0.002 |
| HuatuoGPT-II vs Yuanbao-Hy3 | 3.85 | 0.54 | 0.001 |
| Qwen3.8-Max-Preview vs Yuanbao-Hy3 | 1.04 | 0.15 | 0.374 |

Note. Wilcoxon signed-rank test with Benjamini–Hochberg FDR correction. Z = Wilcoxon Z statistic; r = effect size (Z/√N, where N = 50); r of 0.1, 0.3, and 0.5 correspond to small, medium, and large effects, respectively. Bold values indicate *P*-FDR < 0.05. Overall test: permutation-based Friedman χ² = 13.42, *P* = 0.0001, Kendall’s W = 0.067.

**Table S16.** Pairwise Comparisons for Completeness Using Cumulative Link Mixed Model (CLMM)

| Comparison | CLMM beta (SE) | CLMM OR (95% CI) | CLMM z | CLMM *P*-FDR |
| --- | --- | --- | --- | --- |
| DeepSeek-V4 vs HuatuoGPT-II | 3.957 (0.681) | 52.29 (13.73–199.1) | 5.81 | <0.001 |
| Doubao2.1 vs HuatuoGPT-II | 4.114 (0.699) | 61.20 (15.60–240.2) | 5.88 | <0.001 |
| HuatuoGPT-II vs Qwen3.8-Max-Preview | −4.731 (0.809) | 0.01 (0.002–0.04) | −5.85 | <0.001 |
| HuatuoGPT-II vs Yuanbao-Hy3 | −5.886 (1.158) | 0.003 (0.0003–0.02) | −5.08 | <0.001 |
| DeepSeek-V4 vs Yuanbao-Hy3 | −1.929 (1.111) | 0.15 (0.02–1.22) | −1.74 | 0.165 |
| Doubao2.1 vs Yuanbao-Hy3 | −1.772 (1.127) | 0.17 (0.02–1.54) | −1.57 | 0.193 |
| DeepSeek-V4 vs Qwen3.8-Max-Preview | −0.774 (0.753) | 0.46 (0.11–2.03) | −1.03 | 0.411 |
| Qwen3.8-Max-Preview vs Yuanbao-Hy3 | −1.155 (1.182) | 0.31 (0.03–3.23) | −0.98 | 0.411 |
| Doubao2.1 vs Qwen3.8-Max-Preview | −0.617 (0.776) | 0.54 (0.12–2.46) | −0.80 | 0.474 |
| DeepSeek-V4 vs Doubao2.1 | −0.157 (0.659) | 0.85 (0.24–3.08) | −0.24 | 0.811 |

Note. CLMM = cumulative link mixed model with question ID as random intercept; OR = odds ratio; CI = confidence interval; FDR = Benjamini–Hochberg false discovery rate. Beta > 0 indicates that Model 1 scored higher than Model 2. Bold values indicate *P*-FDR < 0.05. Overall test: likelihood-ratio χ² = 122.47, df = 4, *P* < 0.001; proportional-odds assumption *P* = 0.048 (marginally violated; findings corroborated by non-parametric sensitivity analysis with fully concordant results).

**Table S17.** Pairwise Comparisons for Completeness Using Wilcoxon Signed-Rank Test

| Comparison | Wilcoxon Z | Wilcoxon r | Wilcoxon *P*-FDR |
| --- | --- | --- | --- |
| DeepSeek-V4 vs Doubao2.1 | 0.00 | 0.00 | 1.000 |
| DeepSeek-V4 vs HuatuoGPT-II | 5.49 | 0.78 | <0.001 |
| DeepSeek-V4 vs Qwen3.8-Max-Preview | 1.13 | 0.16 | 0.367 |
| DeepSeek-V4 vs Yuanbao-Hy3 | 1.89 | 0.27 | 0.118 |
| Doubao2.1 vs HuatuoGPT-II | 5.42 | 0.77 | <0.001 |
| Doubao2.1 vs Qwen3.8-Max-Preview | 0.90 | 0.13 | 0.406 |
| Doubao2.1 vs Yuanbao-Hy3 | 1.67 | 0.24 | 0.159 |
| HuatuoGPT-II vs Qwen3.8-Max-Preview | 5.40 | 0.76 | <0.001 |
| HuatuoGPT-II vs Yuanbao-Hy3 | 5.54 | 0.78 | <0.001 |
| Qwen3.8-Max-Preview vs Yuanbao-Hy3 | 1.00 | 0.14 | 0.397 |

Note. Wilcoxon signed-rank test with Benjamini–Hochberg FDR correction. Z = Wilcoxon Z statistic; r = effect size (Z/√N, where N = 50); r of 0.1, 0.3, and 0.5 correspond to small, medium, and large effects, respectively. Bold values indicate *P*-FDR < 0.05. Overall test: permutation-based Friedman χ² = 57.53, *P* = 0.0001, Kendall’s W = 0.288.

**Table S18.** Concordance of significance decisions between cumulative link mixed model and Wilcoxon signed-rank test under FDR correction.

| Dimension | Total comparisons | Concordant | Discordant | Concordance rate |
| --- | --- | --- | --- | --- |
| Accuracy | 10 | 10 | 0 | 100% |
| Caring | 10 | 10 | 0 | 100% |
| Completeness | 10 | 10 | 0 | 100% |
| Overall | 30 | 30 | 0 | 100% |

Note. FDR = Benjamini-Hochberg false discovery rate. Significance was defined as *P*-FDR < 0.05. Under Bonferroni correction, 29 of 30 comparisons were concordant; the single discrepancy occurred in Caring (DeepSeek-V4 vs. Doubao 2.1), where the CLMM result was non-significant whereas the Wilcoxon result remained significant. Under Holm correction, all 30 comparisons were concordant.

**Table S19.**Pairwise *P*-values under Holm correction for all three dimensions.

| Dimension | Comparison | CLMM *P*-raw | CLMM *P*-Holm | Wilcoxon *P*-raw | Wilcoxon *P*-Holm |
| --- | --- | --- | --- | --- | --- |
| Accuracy | DeepSeek-V4 vs HuatuoGPT-II | <0.001 | <0.001 | <0.001 | <0.001 |
| Accuracy | Doubao2.1 vs HuatuoGPT-II | <0.001 | 0.001 | <0.001 | 0.003 |
| Accuracy | HuatuoGPT-II vs Qwen3.8-Max | <0.001 | <0.001 | <0.001 | <0.001 |
| Accuracy | HuatuoGPT-II vs Yuanbao-Hy3 | <0.001 | <0.001 | <0.001 | 0.001 |
| Accuracy | DeepSeek-V4 vs Doubao2.1 | 0.108 | 0.646 | 0.102 | 0.615 |
| Accuracy | DeepSeek-V4 vs Qwen3.8-Max | 0.212 | 1 | 0.157 | 0.786 |
| Accuracy | DeepSeek-V4 vs Yuanbao-Hy3 | 0.371 | 1 | 0.371 | 1 |
| Accuracy | Doubao2.1 vs Qwen3.8-Max | 0.711 | 1 | 0.67 | 1 |
| Accuracy | Doubao2.1 vs Yuanbao-Hy3 | 0.464 | 1 | 0.371 | 1 |
| Accuracy | Qwen3.8-Max vs Yuanbao-Hy3 | 0.717 | 1 | 0.683 | 1 |
| Caring | DeepSeek-V4 vs HuatuoGPT-II | <0.001 | <0.001 | <0.001 | 0.002 |
| Caring | HuatuoGPT-II vs Yuanbao-Hy3 | <0.001 | <0.001 | <0.001 | 0.001 |
| Caring | HuatuoGPT-II vs Qwen3.8-Max | <0.001 | 0.002 | <0.001 | 0.006 |
| Caring | DeepSeek-V4 vs Doubao2.1 | 0.007 | 0.044 | 0.003 | 0.018 |
| Caring | Doubao2.1 vs Yuanbao-Hy3 | 0.003 | 0.02 | 0.003 | 0.018 |
| Caring | Doubao2.1 vs HuatuoGPT-II | 0.026 | 0.131 | 0.025 | 0.127 |
| Caring | Doubao2.1 vs Qwen3.8-Max | 0.071 | 0.283 | 0.048 | 0.192 |
| Caring | Qwen3.8-Max vs Yuanbao-Hy3 | 0.212 | 0.635 | 0.299 | 0.897 |
| Caring | DeepSeek-V4 vs Qwen3.8-Max | 0.364 | 0.728 | 0.39 | 0.897 |
| Caring | DeepSeek-V4 vs Yuanbao-Hy3 | 0.724 | 0.728 | 0.864 | 0.897 |
| Completeness | DeepSeek-V4 vs HuatuoGPT-II | <0.001 | <0.001 | <0.001 | <0.001 |
| Completeness | Doubao2.1 vs HuatuoGPT-II | <0.001 | <0.001 | <0.001 | <0.001 |
| Completeness | HuatuoGPT-II vs Qwen3.8-Max | <0.001 | <0.001 | <0.001 | <0.001 |
| Completeness | HuatuoGPT-II vs Yuanbao-Hy3 | <0.001 | <0.001 | <0.001 | <0.001 |
| Completeness | DeepSeek-V4 vs Yuanbao-Hy3 | 0.082 | 0.495 | 0.059 | 0.353 |
| Completeness | Doubao2.1 vs Yuanbao-Hy3 | 0.116 | 0.579 | 0.096 | 0.478 |
| Completeness | DeepSeek-V4 vs Qwen3.8-Max | 0.304 | 1 | 0.257 | 1 |
| Completeness | Qwen3.8-Max vs Yuanbao-Hy3 | 0.329 | 1 | 0.317 | 1 |
| Completeness | Doubao2.1 vs Qwen3.8-Max | 0.427 | 1 | 0.366 | 1 |
| Completeness | DeepSeek-V4 vs Doubao2.1 | 0.811 | 1 | 1 | 1 |

**Table S20.**Pairwise *P*-values under Bonferroni correction for all three dimensions.

| Dimension | Comparison | CLMM *P*-Bonferroni | Wilcoxon *P*-Bonferroni |
| --- | --- | --- | --- |
| Accuracy | DeepSeek-V4 vs HuatuoGPT-II | <0.001 | <0.001 |
| Accuracy | Doubao2.1 vs HuatuoGPT-II | 0.001 | 0.004 |
| Accuracy | HuatuoGPT-II vs Qwen3.8-Max | <0.001 | <0.001 |
| Accuracy | HuatuoGPT-II vs Yuanbao-Hy3 | <0.001 | 0.002 |
| Accuracy | DeepSeek-V4 vs Doubao2.1 | 1 | 1 |
| Accuracy | DeepSeek-V4 vs Qwen3.8-Max | 1 | 1 |
| Accuracy | DeepSeek-V4 vs Yuanbao-Hy3 | 1 | 1 |
| Accuracy | Doubao2.1 vs Qwen3.8-Max | 1 | 1 |
| Accuracy | Doubao2.1 vs Yuanbao-Hy3 | 1 | 1 |
| Accuracy | Qwen3.8-Max vs Yuanbao-Hy3 | 1 | 1 |
| Caring | DeepSeek-V4 vs HuatuoGPT-II | <0.001 | 0.002 |
| Caring | HuatuoGPT-II vs Yuanbao-Hy3 | <0.001 | 0.001 |
| Caring | HuatuoGPT-II vs Qwen3.8-Max | 0.002 | 0.007 |
| Caring | DeepSeek-V4 vs Doubao2.1 | 0.074 | 0.029 |
| Caring | Doubao2.1 vs Yuanbao-Hy3 | 0.028 | 0.026 |
| Caring | Doubao2.1 vs HuatuoGPT-II | 0.263 | 0.254 |
| Caring | Doubao2.1 vs Qwen3.8-Max | 0.708 | 0.48 |
| Caring | Qwen3.8-Max vs Yuanbao-Hy3 | 1 | 1 |
| Caring | DeepSeek-V4 vs Qwen3.8-Max | 1 | 1 |
| Caring | DeepSeek-V4 vs Yuanbao-Hy3 | 1 | 1 |
| Completeness | DeepSeek-V4 vs HuatuoGPT-II | <0.001 | <0.001 |
| Completeness | Doubao2.1 vs HuatuoGPT-II | <0.001 | <0.001 |
| Completeness | HuatuoGPT-II vs Qwen3.8-Max | <0.001 | <0.001 |
| Completeness | HuatuoGPT-II vs Yuanbao-Hy3 | <0.001 | <0.001 |
| Completeness | DeepSeek-V4 vs Yuanbao-Hy3 | 0.824 | 0.588 |
| Completeness | Doubao2.1 vs Yuanbao-Hy3 | 1 | 0.956 |
| Completeness | DeepSeek-V4 vs Qwen3.8-Max | 1 | 1 |
| Completeness | Qwen3.8-Max vs Yuanbao-Hy3 | 1 | 1 |
| Completeness | Doubao2.1 vs Qwen3.8-Max | 1 | 1 |
| Completeness | DeepSeek-V4 vs Doubao2.1 | 1 | 1 |

**Table S21.** Overall Differences and Significant Pairwise Comparisons for Understandability

| Item / Total Score | Overall Difference | Significant Pairwise Comparisons (FDR-adjusted) |
| --- | --- | --- |
| Item 1: Purpose evident | No (*P* = 0.349, W = 0.022) | None |
| Item 2: Content focus | No (*P* = 0.406, W = 0.020) | None |
| Item 3: Common language | Yes (*P* = 0.002, W = 0.084) | DeepSeek-V4 vs. Yuanbao-Hy3 (*P* = 0.009, OR = 5.50) |
| Item 4: Medical terms defined | No (*P* = 0.080, W = 0.042) | None |
| Item 5: Information chunking | Yes (*P* < 0.001, W = 0.260) | DeepSeek-V4 vs. HuatuoGPT-II (*P* = 0.002, OR = ∞); Doubao2.1 vs. HuatuoGPT-II (*P* = 0.002, OR = ∞); HuatuoGPT-II vs. Qwen3.8-Max-Preview (*P* = 0.002, OR = 0); HuatuoGPT-II vs. Yuanbao-Hy3 (*P* = 0.002, OR = 0) |
| Item 6: Logical organization and clear flow | No (*P* = 0.406, W = 0.020) | None |
| Item 7: Visual cue effectiveness | Yes (*P* < 0.001, W = 0.280) | DeepSeek-V4 vs. HuatuoGPT-II (*P* = 0.001, OR = ∞); Doubao2.1 vs. HuatuoGPT-II (*P* = 0.001, OR = ∞); HuatuoGPT-II vs. Qwen3.8-Max-Preview (*P* = 0.001, OR = 0); HuatuoGPT-II vs. Yuanbao-Hy3 (*P*= 0.001, OR = 0) |
| Understandability Total (0–7) | Yes (*P* = 0.004, W = 0.077) | DeepSeek-V4 vs. Doubao2.1 (*P* = 0.047, r = 0.347); DeepSeek-V4 vs. HuatuoGPT-II (*P* = 0.001, r = 0.546); DeepSeek-V4 vs. Yuanbao-Hy3 (*P* = 0.022, r = 0.403) |

Note. Overall differences for individual items were assessed using Cochran’s Q test; the total score was assessed using the Friedman test. W denotes Kendall’s coefficient of concordance. Pairwise comparisons used McNemar’s test (individual items) or Wilcoxon signed-rank test (total score), with Benjamini–Hochberg FDR correction. OR = odds ratio; r = effect size (Z/√N). OR = ∞ or 0 indicates all discordant pairs fell in one direction. Results under Holm and Bonferroni corrections are provided in Supplementary Table S22.

**Table S22.** Pairwise comparisons for Understandability items and total score under Holm and Bonferroni corrections.

| Item / Total Score | Pairwise Comparison | Effect Size (OR / r) | Holm-adjusted *P* | Sig. (Holm) | Bonferroni-adjusted *P* | Sig. (Bonferroni) |
| --- | --- | --- | --- | --- | --- | --- |
| Item 3: Common language | DeepSeek-V4 vs. Doubao2.1 | OR = 3.40 | 0.171 | No | 0.190 | No |
|  | DeepSeek-V4 vs. HuatuoGPT-II | OR = 2.00 | 1.000 | No | 1.000 | No |
|  | DeepSeek-V4 vs. Qwen3.8-Max-Preview | OR = 2.00 | 1.000 | No | 1.000 | No |
|  | DeepSeek-V4 vs. Yuanbao-Hy3 | OR = 5.50 | 0.009 | Yes | 0.009 | Yes |
|  | Doubao2.1 vs. HuatuoGPT-II | OR = 0.54 | 1.000 | No | 1.000 | No |
|  | Doubao2.1 vs. Qwen3.8-Max-Preview | OR = 0.64 | 1.000 | No | 1.000 | No |
|  | Doubao2.1 vs. Yuanbao-Hy3 | OR = 1.67 | 1.000 | No | 1.000 | No |
|  | HuatuoGPT-II vs. Qwen3.8-Max-Preview | OR = 1.13 | 1.000 | No | 1.000 | No |
|  | HuatuoGPT-II vs. Yuanbao-Hy3 | OR = 2.71 | 0.233 | No | 0.310 | No |
|  | Qwen3.8-Max-Preview vs. Yuanbao-Hy3 | OR = 3.20 | 0.233 | No | 0.291 | No |
| Item 5: Information chunking | DeepSeek-V4 vs. HuatuoGPT-II | OR = ∞ | 0.009 | Yes | 0.009 | Yes |
|  | Doubao2.1 vs. HuatuoGPT-II | OR = ∞ | 0.009 | Yes | 0.009 | Yes |
|  | HuatuoGPT-II vs. Qwen3.8-Max-Preview | OR = 0 | 0.009 | Yes | 0.009 | Yes |
|  | HuatuoGPT-II vs. Yuanbao-Hy3 | OR = 0 | 0.009 | Yes | 0.009 | Yes |
|  | All other pairs | OR = 1.00 | 1.000 | No | 1.000 | No |
| Item 7: Visual cues | DeepSeek-V4 vs. HuatuoGPT-II | OR = ∞ | 0.005 | Yes | 0.005 | Yes |
|  | Doubao2.1 vs. HuatuoGPT-II | OR = ∞ | 0.005 | Yes | 0.005 | Yes |
|  | HuatuoGPT-II vs. Qwen3.8-Max-Preview | OR = 0 | 0.005 | Yes | 0.005 | Yes |
|  | HuatuoGPT-II vs. Yuanbao-Hy3 | OR = 0 | 0.005 | Yes | 0.005 | Yes |
|  | All other pairs | OR = 1.00 | 1.000 | No | 1.000 | No |
| Understandability Total | DeepSeek-V4 vs. Doubao2.1 | r = 0.347 | 0.113 | No | 0.142 | No |
|  | DeepSeek-V4 vs. HuatuoGPT-II | r = 0.546 | 0.001 | Yes | 0.001 | Yes |
|  | DeepSeek-V4 vs. Qwen3.8-Max-Preview | r = 0.306 | 0.215 | No | 0.307 | No |
|  | DeepSeek-V4 vs. Yuanbao-Hy3 | r = 0.403 | 0.039 | Yes | 0.043 | Yes |
|  | Doubao2.1 vs. HuatuoGPT-II | r = 0.229 | 0.526 | No | 1.000 | No |
|  | Doubao2.1 vs. Qwen3.8-Max-Preview | r = 0.055 | 0.951 | No | 1.000 | No |
|  | Doubao2.1 vs. Yuanbao-Hy3 | r = 0.101 | 0.951 | No | 1.000 | No |
|  | HuatuoGPT-II vs. Qwen3.8-Max-Preview | r = 0.296 | 0.218 | No | 0.364 | No |
|  | HuatuoGPT-II vs. Yuanbao-Hy3 | r = 0.197 | 0.656 | No | 1.000 | No |
|  | Qwen3.8-Max-Preview vs. Yuanbao-Hy3 | r = 0.183 | 0.656 | No | 1.000 | No |

Note. Items 1, 2, 4, and 6 showed no significant overall difference (Cochran's Q, all *P* > 0.05); pairwise comparisons were therefore not conducted. Bold values indicate statistical significance at α = 0.05.

**Table S23.** Overall Differences and Significant Pairwise Comparisons for Actionability

| Item / Total Score | Overall Difference | Significant Pairwise Comparisons (FDR-adjusted) |
| --- | --- | --- |
| Item 8: Action identified | No (*P* = 0.273, W = 0.026) | None |
| Item 9: Direct address | Yes (*P* < 0.001, W = 0.144) | DeepSeek-V4 vs. Doubao2.1 (*P* = 0.001, OR = 19.00); DeepSeek-V4 vs. Qwen3.8-Max-Preview (*P* = 0.008, OR = 6.00); Doubao2.1 vs. HuatuoGPT-II (*P* = 0.019, OR = 0); Doubao2.1 vs. Yuanbao-Hy3 (*P* = 0.004, OR = 0); Qwen3.8-Max-Preview vs. Yuanbao-Hy3 (*P*= 0.019, OR = 0.09) |
| Item 10: Manageable steps | Yes (*P* < 0.001, W = 0.142) | DeepSeek-V4 vs. HuatuoGPT-II (*P* < 0.001, OR = 21.00); Doubao2.1 vs. HuatuoGPT-II (*P* = 0.015, OR = 5.33); HuatuoGPT-II vs. Qwen3.8-Max-Preview (*P* = 0.012, OR = 0.18); HuatuoGPT-II vs. Yuanbao-Hy3 (*P* < 0.001, OR = 0.05) |
| Actionability Total (0–3) | Yes (*P* < 0.001, W = 0.133) | DeepSeek-V4 vs. Doubao2.1 (*P*= 0.003, r = 0.460); DeepSeek-V4 vs. HuatuoGPT-II (*P* = 0.003, r = 0.475); DeepSeek-V4 vs. Qwen3.8-Max-Preview (*P* = 0.018, r = 0.370); Doubao2.1 vs. Yuanbao-Hy3 (*P* = 0.002, r = 0.504); HuatuoGPT-II vs. Yuanbao-Hy3 (*P* = 0.002, r = 0.509); Qwen3.8-Max-Preview vs. Yuanbao-Hy3 (*P* = 0.023, r = 0.348) |

Note. Overall differences for individual items were assessed using Cochran’s Q test; the total score was assessed using the Friedman test. W denotes Kendall’s coefficient of concordance. Pairwise comparisons used McNemar’s test (individual items) or Wilcoxon signed-rank test (total score), with Benjamini–Hochberg FDR correction. OR = odds ratio; r = effect size (Z/√N). OR = ∞ or 0 indicates all discordant pairs fell in one direction. Results under Holm and Bonferroni corrections are provided in Supplementary Table S24.

**Table S24.** Pairwise comparisons for Actionability items and total score under Holm and Bonferroni corrections.

| Item / Total Score | Pairwise Comparison | Effect Size (OR / r) | Holm-adjusted *P* | Sig. (Holm) | Bonferroni-adjusted *P* | Sig. (Bonferroni) |
| --- | --- | --- | --- | --- | --- | --- |
| Item 9: Direct address | DeepSeek-V4 vs. Doubao2.1 | OR = 19.00 | 0.001 | Yes | 0.001 | Yes |
|  | DeepSeek-V4 vs. HuatuoGPT-II | OR = 2.50 | 0.385 | No | 0.809 | No |
|  | DeepSeek-V4 vs. Qwen3.8-Max-Preview | OR = 6.00 | 0.018 | Yes | 0.023 | Yes |
|  | DeepSeek-V4 vs. Yuanbao-Hy3 | OR = 1.50 | 1.000 | No | 1.000 | No |
|  | Doubao2.1 vs. HuatuoGPT-II | OR = 0 | 0.054 | No | 0.077 | No |
|  | Doubao2.1 vs. Qwen3.8-Max-Preview | OR = 0.25 | 1.000 | No | 1.000 | No |
|  | Doubao2.1 vs. Yuanbao-Hy3 | OR = 0 | 0.008 | Yes | 0.009 | Yes |
|  | HuatuoGPT-II vs. Qwen3.8-Max-Preview | OR = 7.00 | 0.385 | No | 0.771 | No |
|  | HuatuoGPT-II vs. Yuanbao-Hy3 | OR = 0.56 | 1.000 | No | 1.000 | No |
|  | Qwen3.8-Max-Preview vs. Yuanbao-Hy3 | OR = 0.09 | 0.056 | No | 0.094 | No |
| Item 10: Manageable steps | DeepSeek-V4 vs. Doubao2.1 | OR = 2.40 | 0.874 | No | 1.000 | No |
|  | DeepSeek-V4 vs. HuatuoGPT-II | OR = 21.00 | 0.001 | Yes | 0.001 | Yes |
|  | DeepSeek-V4 vs. Qwen3.8-Max-Preview | OR = 2.20 | 1.000 | No | 1.000 | No |
|  | DeepSeek-V4 vs. Yuanbao-Hy3 | OR = 1.11 | 1.000 | No | 1.000 | No |
|  | Doubao2.1 vs. HuatuoGPT-II | OR = 5.33 | 0.041 | Yes | 0.059 | No |
|  | Doubao2.1 vs. Qwen3.8-Max-Preview | OR = 0.86 | 1.000 | No | 1.000 | No |
|  | Doubao2.1 vs. Yuanbao-Hy3 | OR = 0.45 | 1.000 | No | 1.000 | No |
|  | HuatuoGPT-II vs. Qwen3.8-Max-Preview | OR = 0.18 | 0.029 | Yes | 0.037 | Yes |
|  | HuatuoGPT-II vs. Yuanbao-Hy3 | OR = 0.05 | 0.001 | Yes | 0.001 | Yes |
|  | Qwen3.8-Max-Preview vs. Yuanbao-Hy3 | OR = 0.55 | 1.000 | No | 1.000 | No |
| Actionability Total | DeepSeek-V4 vs. Doubao2.1 | r = 0.460 | 0.008 | Yes | 0.011 | Yes |
|  | DeepSeek-V4 vs. HuatuoGPT-II | r = 0.475 | 0.006 | Yes | 0.008 | Yes |
|  | DeepSeek-V4 vs. Qwen3.8-Max-Preview | r = 0.370 | 0.054 | No | 0.089 | No |
|  | DeepSeek-V4 vs. Yuanbao-Hy3 | r = 0.084 | 1.000 | No | 1.000 | No |
|  | Doubao2.1 vs. HuatuoGPT-II | r = 0.042 | 1.000 | No | 1.000 | No |
|  | Doubao2.1 vs. Qwen3.8-Max-Preview | r = 0.214 | 0.522 | No | 1.000 | No |
|  | Doubao2.1 vs. Yuanbao-Hy3 | r = 0.504 | 0.003 | Yes | 0.004 | Yes |
|  | HuatuoGPT-II vs. Qwen3.8-Max-Preview | r = 0.180 | 0.608 | No | 1.000 | No |
|  | HuatuoGPT-II vs. Yuanbao-Hy3 | r = 0.509 | 0.003 | Yes | 0.003 | Yes |
|  | Qwen3.8-Max-Preview vs. Yuanbao-Hy3 | r = 0.348 | 0.069 | No | 0.139 | No |

Note. Item 8 (Action identified) showed no significant overall difference (Cochran's Q, *P* = 0.273); pairwise comparisons were therefore not conducted. Bold values indicate statistical significance at α = 0.05. OR = odds ratio from McNemar's test; r = effect size from Wilcoxon signed-rank test.

**Table S25.** Between-group comparisons of the four latent profiles across quality dimensions.

| Variable | F | *P*(ANOVA) | H | *P* (KW) |
| --- | --- | --- | --- | --- |
| Accuracy | 160.16 | <.001 | 194.75 | <.001 |
| Caring | 10.29 | <.001 | 33.23 | <.001 |
| Completeness | 260.61 | <.001 | 239.53 | <.001 |
| Understandability | 1.58 | .194 | 2.64 | .451 |
| Actionability | 4.20 | .006 | 13.30 | .004 |

Note. F = one-way ANOVA; H = Kruskal–Wallis test. *P*(ANOVA) and *P*(KW) are the corresponding *P* values. Profile labels are as defined in Table 7. Understandability did not differ significantly across profiles (ANOVA p = .194; Kruskal–Wallis p = .451), indicating a ceiling effect in readability across all AI models.

**Table S26** Regularized Partial Correlation Matrix Among Five AI Response Quality Dimensions Estimated via Graphical Lasso

|  | Accuracy | Completeness | Understandability | Actionability | Caring |
| --- | --- | --- | --- | --- | --- |
| Accuracy | — | 0.490 | 0 | −0.083 | 0.111 |
| Completeness | 0.490 | — | 0.083 | 0.112 | 0.127 |
| Understandability | 0 | 0.083 | — | 0.067 | 0 |
| Actionability | −0.083 | 0.112 | 0.067 | — | 0.233 |
| Caring | 0.111 | 0.127 | 0 | 0.233 | — |

Note. Partial correlations estimated via Graphical Lasso (λ selected by 5-fold CV); values below |0.05| set to zero. The strongest edge was Accuracy–Completeness (r = 0.490), followed by Actionability–Caring (r = 0.233).

**Table S27.**Robustness Checks for Question-Level Dependency in LPA and Network Analyses.

| Strategy | Data Characteristics | LPA Results | Network Edge Weight Concordance (Pearson r) | Centrality Ranking Concordance (Spearman ρ) |
| --- | --- | --- | --- | --- |
| **Primary analysis** | N = 250 (5 models × 50 questions) | K = 4 (Entropy = 0.998, MinAvePP = 0.999, ARI = 0.823) | — | — |
| **Question-mean centering** | N = 250 (question main effects removed) | K = 4 (profiles morphologically similar to primary) | **0.942** | **0.900** |
| **Question-level aggregation** | N = 50 (mean score per question across 5 models) | **K = 1** (single-class solution) | — | — |

Note: ARI = Adjusted Rand Index based on 200 bootstrap resamples; Entropy > 0.99 and MinAvePP > 0.99 indicate excellent classification quality.
